# Challenging a paradigm: Staggered versus single-pulse mass dog vaccination strategy for rabies elimination

**DOI:** 10.1371/journal.pcbi.1012780

**Published:** 2025-02-07

**Authors:** Brinkley Raynor Bellotti, Elvis W. Díaz, Micaela De la Puente-León, Maria T. Rieders, Sergio E. Recuenco, Michael Z. Levy, Ricardo Castillo-Neyra

**Affiliations:** 1 Department of Biostatistics, Epidemiology, and Informatics, University of Pennsylvania, Philadelphia, Pennsylvania, United States of America; 2 Section on Infectious Diseases, Wake Forest University School of Medicine, Winston-Salem, North Carolina, United States of America; 3 Zoonotic Disease Research Lab, One Health Unit, School of Public Health and Administration, Universidad Peruana Cayetano Heredia, Lima, Perú; 4 Operations, Information and Decisions Department, University of Pennsylvania, Philadelphia, Pennsylvania, United States of America; 5 Department of Preventive Medicine and Public Health, Faculty of Medicine San Fernando, Universidad Nacional Mayor de San Marcos, Lima, Peru; Animal and Plant Health Inspection Service, UNITED STATES OF AMERICA

## Abstract

**Background:**

From smallpox to poliomyelitis, halting contagion transmission through simultaneous mass vaccination is ubiquitous and often perceived as the only possible solution. But implementing mass vaccination campaigns in large populations within a short period poses many challenges. For example, in Arequipa, Peru, sweeping mass vaccination campaigns conducted yearly over a single weekend have failed to achieve the required ‘herd immunity’ to halt canine rabies transmission. Contrary to the global paradigm of a simultaneous campaign, the 2022 Arequipa rabies campaign was implemented at the sub-district level (patches), with dates of the campaign staggered across 6 months.

**Methods:**

We constructed a stochastic, metapopulation model to examine how the timing of pulsed vaccination campaigns across patches can affect metapopulation dynamics. We explore general metapopulation dynamics for pulsed vaccinations as well as parameterizing the model for canine rabies in Arequipa, Peru. We simulated how the timing of the planned vaccination campaign, staggered over 6 months versus a single yearly pulse, affected the prospects for regional rabies elimination.

**Results:**

Metapopulation dynamics can affect the efficacy of pulsed vaccination campaigns. In the case of Arequipa, Peru, the planned staggered mass dog vaccination campaign has the potential for local elimination with the tradeoffs of increased time to elimination and increased outbreak size due to metapopulation dynamics.

**Conclusions:**

Heterogeneities caused by control strategies enactment at sub-population scales should be accounted for when modeling transmission dynamics. In Arequipa, Peru, although metapopulation dynamics may allow for re-introduction of canine rabies in previously vaccinated patches when mass dog vaccination campaigns are staggered temporally over 6 months, continuous mass vaccination reaching recommended vaccination coverage levels is sufficient to eliminate canine rabies.

## Author summary

Metapopulation models that incorporate local variability in control measure implementation and population dynamics offer valuable insights for policymaking. The current paradigm for dog rabies elimination involves “single-pulse” mass dog vaccination campaigns where the campaign is conducted within a short period (single day or weekend). While historically effective, single-pulse strategies pose significant feasibility challenges in large populations. For example, Arequipa, Peru, a city with an ongoing rabies outbreak among the free-roaming dog population, has a dog population of approximately 250,000. We constructed metapopulation models to explore how extending timing of vaccination campaigns over a period of months (staggered-pulse) focusing on sequential sub-regions would affect disease control prospects. Our models show that a staggered mass dog vaccination campaign that achieves herd immunity could effectively eliminate canine rabies in scenarios where achieving adequate coverage within a brief period is unfeasible. Our results suggest that a shifting paradigm that allows longer vaccination campaigns to achieve sufficient coverage can benefit both local policymakers and the worldwide goal of ‘zero human deaths by 2030’.

## Introduction

Dogs are the source of approximately 99% of human rabies cases [1]; the World Health Organization has set a ‘Zero by Thirty’ goal aiming for zero human deaths globally from dog-mediated rabies by 2030 [1]. The mainstay of rabies control and elimination is mass dog vaccination. The ideal mass vaccination campaign would vaccinate 70–80% [2–5] of the entire dog population instantly. This vaccination ‘pulse’ would theoretically have the effect of eliminating rabies, similar to throwing a wet blanket over a fire, by conferring herd immunity sufficient to break the chain of dog-to-dog rabies virus transmission [2,5,6]. In the Americas, these pulsed campaign types were successful at eliminating polio and measles [6]. However, as instantaneous vaccination of large numbers of dogs is impossible, rabies elimination programs aim to vaccinate as many dogs as possible as quickly as possible [5,7]. The fear of a temporally drawn-out vaccination campaign is that rapid dog population turnover will allow persistence of rabies virus due to replenishment of the susceptible population as unvaccinated puppies replace older dogs dying of non-rabies related causes [8–10]. There is little literature on how the timing of pulse vaccination campaigns among canine subpopulations affects rabies virus transmission dynamics [10].

Rabies transmission dynamics are generally modeled using an SEIV (susceptible - exposed - infected - vaccinated) compartment model framework [9,11–15]. In simplified models, homogeneity of the dog population is assumed. However, in some cases, models that account for population heterogeneity are required to address the research topic of interest. Specifically, metapopulation models can address population variation due to geopolitical boundaries (patches), such as different policies in different boundaries [16–19]. Metapopulation models with continuous vaccination are well characterized in the rabies literature [9,14] and general SIR (susceptible-infectious-resistant) metapopulation models with pulsed vaccination have been characterized in the literature [20,21], but there has been little work on rabies metapopulation models with pulsed vaccination strategies. Keeling and Rohani explored transmission dynamics non-specific to rabies with synchronous and asynchronous vaccination pulses in different patches, finding that metapopulation dynamics can lead to unexpected elimination prospects due to the degree of coupling between patches and subsequent synchrony of dynamics [20]. Terry presented a framework for SIR metapopulation models with an analytic derivation of the conditions in which elimination would occur in different vaccination scenarios [21].

Currently, in Arequipa, there are estimated to be approximately 250,000 owned dogs [22]. Due to feasibility constraints, in 2022, the mass vaccination plan transitioned from a citywide single pulse vaccination to a multi-month roll out vaccinating different *microreds* (sub-district geographic units) each weekend. However, due to the rapid rate of population turnover [13] and the spatial connectivity of the *microreds*, public health officials are concerned about the long-term success of this vaccination strategy. We aim to quantify the prospects of canine rabies elimination using a staggered pulse mass vaccination strategy through metapopulation model simulations.

## Methods

### Theoretical model

#### Model formulation.

**Fig 1:**
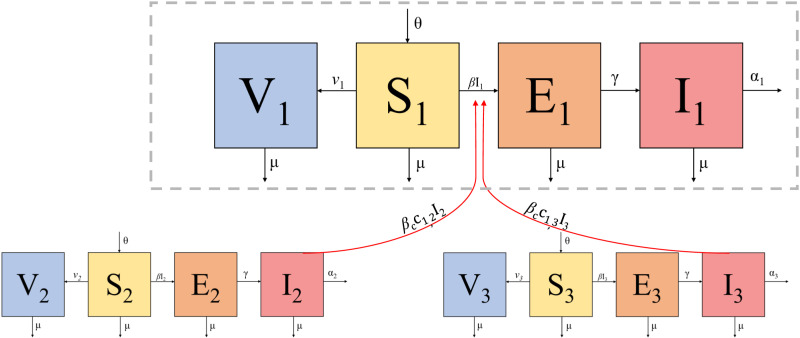
Flow diagram of patch dynamics. Within each patch, each sub-population follows SEIV dynamics. A single patch with-in patch dynamics is represented within the dotted gray box. This example depicts three patches with the gray box encapsulating patch 1. In addition to the within-patch dynamics, red arrows indicate additional transmission pathways, representing infected individuals from patches 2 and 3 exposing susceptible from patch 1.


To explore the dynamics of diseases in response to spatially staggered pulse vaccination campaigns, we constructed deterministic models metapopulation (patch) models with pulsed mass vaccination independent per patch accompanied by stochastic iterations. Patches in the model represent sub-district geographic units at which vaccination public health policies are implemented. Derivations and code followed the method presented in McCormack and Allen, 2006 with the addition of an ‘exposed’ compartment. Briefly, a flexible system of interacting stochastic differential equations (SDE’s) was constructed where each patch has within cell susceptible-exposed-infectious-vaccinated (SEIV) dynamics (Fig 1), with exchange of infectious individuals between patches (Fig 1, Equations 1.1–1.4) [23,24]. Vaccination rates are modeled as a time-varying parameter so that rates can be pulsed in accordance with vaccination campaign dates. Stochastic variability is incorporated into birth, death, disease transmission, and incubation processes. Stochastic variability is normally distributed over the following system of ODE’s:

$$dS_{i}=\theta -\beta S_{i}I_{i}/N_{i}-\nu S_{i}-\mu S_{i\;}-\sum_{1}^{j}\beta_{C}\;c\;I_{j}S_{i}/N_{i}$$ (1.1)

$$dE_{i}=\beta S_{i}I_{i}/N_{i}-\gamma E_{i}-\mu E_{i}\;+\sum_{1}^{j}\beta_{C}\;c\;I_{j}S_{i}/N_{i}$$ (1.2)

$$dI_{i}=\gamma E_{i}-\alpha I_{i}-\mu I_{i}$$ (1.3)

$$dV_{i}=\nu S_{i}-\mu V_{i}$$ (1.4)

where bolded state variables (**S**, **E**, **I**, **V**) indicate vectors of length *j* of the disease states Susceptible, Exposed, Infectious, and Vaccinated; *j* is the number of sub-populations (patches) in the system. The total population size, **N**, is the sum of the state variables. Bolded parameters (*ν*, *α*) indicate vectors of length *j* where each entry is the estimated vaccination rate for each patch ($\nu_{j}$) and death rate attributable to rabies (*α*_j_ ). The bolded **c** denotes a contact matrix where the *i, j*^th^ element of the matrix indicates the normalized contact rates between patches *i* and *j.* Other parameters are assumed to be equal across patches in the deterministic formulation and from the same distributions in the stochastic iterations, with *θ* indicating the birth rate, *β* indicating the within-patch transmission coefficient, $\beta_{c}$ indicating the between-patch transmission coefficient scalar, *γ* indicating the incubation rate, and *μ* indicating the background death rate. As an example, patch 1 is governed by Equations 2.1–2.4 (Fig 1).


$$d{\text{S}}_{1}=\theta -\beta {\text{S}}_{1}{\text{I}}_{1}/{\text{N}}_{1}-(\beta_{C}{\text{c}}_{1,2}{\text{S}}_{1}{\text{I}}_{2}/{\text{N}}_{1}+...+\beta_{C}{\text{c}}_{1,\text{n}}{\text{S}}_{1}{\text{I}}_{\text{n}}/{\text{N}}_{1})-\nu_{1}{\text{S}}_{1}-\mu {\text{S}}_{1}$$ (2.1)

$$d{\text{E}}_{1}=\beta {\text{S}}_{1}{\text{I}}_{1}/{\text{N}}_{1}+(\beta_{C}{\text{c}}_{1,2}{\text{S}}_{1}{\text{I}}_{2}/{\text{N}}_{1}+\;...+\beta_{C}{\text{c}}_{1,\text{n}}{\text{S}}_{1}{\text{I}}_{\text{n}}/{\text{N}}_{1})-\gamma {\text{E}}_{1}-\mu {\text{E}}_{1}$$ (2.2)

$$d{\text{I}}_{1}=\gamma {\text{E}}_{1}-{\text{α}}_{1}{\text{I}}_{1}-\mu {\text{I}}_{1}$$ (2.3)

$$d{\text{V}}_{1}=\nu_{1}{\text{S}}_{1}-\mu {\text{V}}_{1}$$ (2.4)

#### Forward simulation.

**Fig 2:**
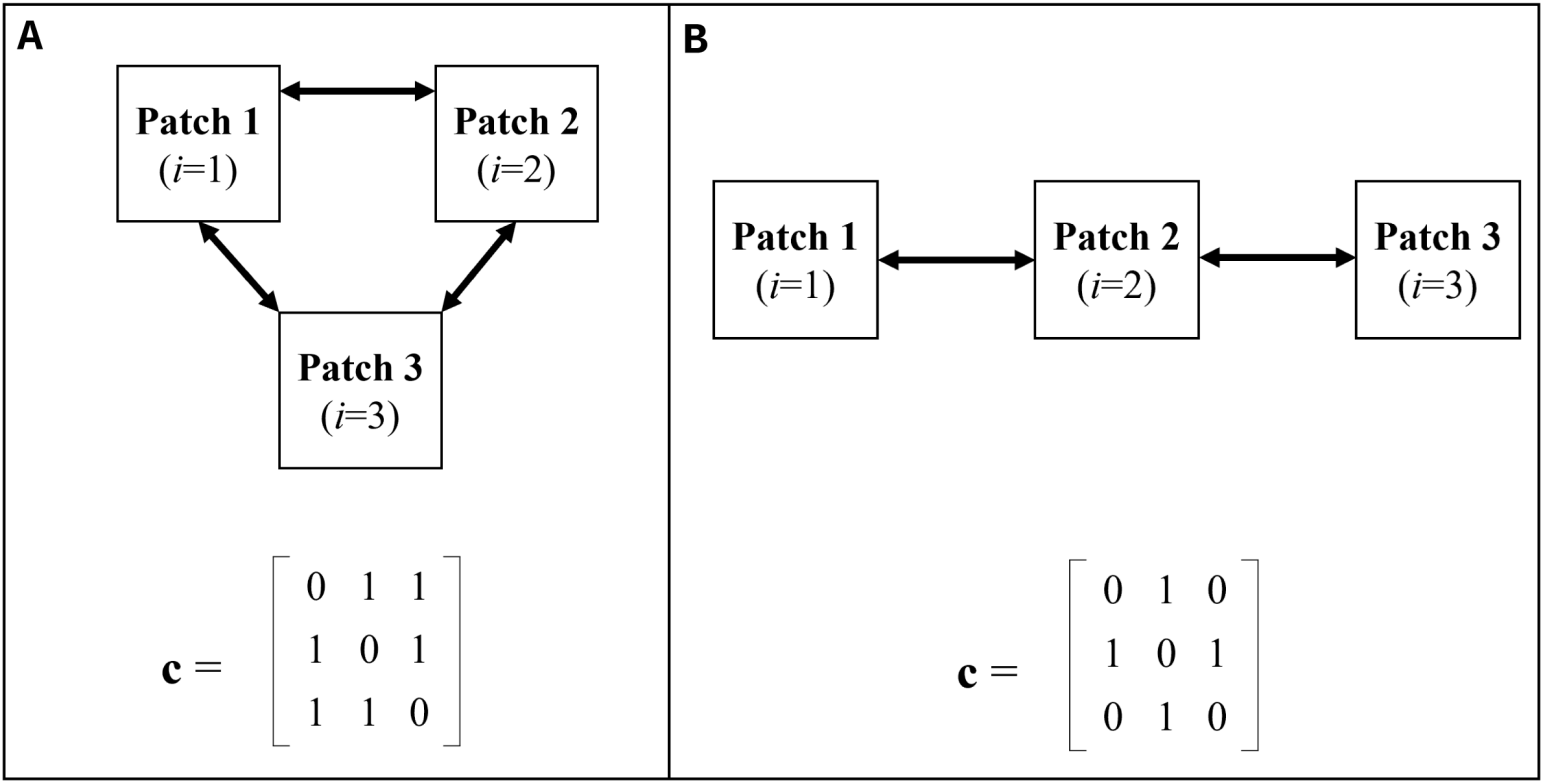
Two theoretical 3-patch metapopulation structures with their corresponding contact matrices, c. In the fully connected system (A), all of the patches interact with each other equally. In the semi-connected system (B), patch 2 interacts with both patches 1 and 3, but 1 and 3 do not interact with each other.

We ran model simulations for two simplified 3-patch models, one where all patches were connected equally (Fig 2A) and one where they were connected unequally (Fig 2B) in order to demonstrate dynamics unique to metapopulation SEIV with pulsed vaccination. We ran a series of vaccination strategies with hypothetical parameters to reflect a general, hypothetical disease (see S1 Text for full details). In this hypothetical, simplified system, the same level of vaccination (70%) was achieved in all simulation scenarios. The single pulse campaign strategy was timed so that all patches were vaccinated on the same day. The staggered campaign was timed over a 100-day time period (a patch getting vaccinated, the next patch getting vaccinated 50 days later, and the last patch getting vaccinated 50 days after that).

### Rabies model

**Fig 3:**
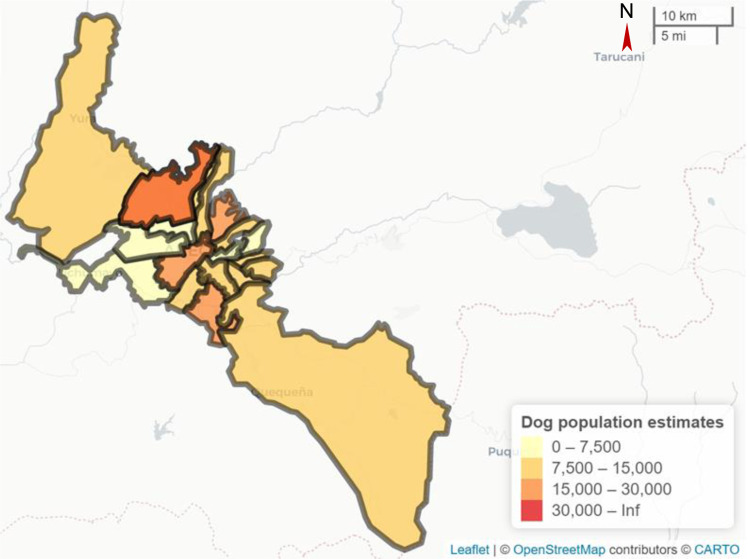
Map of *microreds* in Arequipa, colored by dog population size. Dog population estimates are from the Ministry of Health [20]. Maps were created using the Leaflet R package [25]. Base map (https://carto.com/basemaps) and data from OpenStreetMap and OpenStreetMap Foundation [26]. The microred borders were drawn in collaboration with the Arequipa Department of Health, spatial data available at https://github.com/bhraynor/RabiesPatchVax.

Adapting the theoretical model for canine rabies in Arequipa, patches in the model represent microreds, the sub-district geographic unit at which vaccination campaigns are implemented in Arequipa (Fig 3). Immigration and emigration of dogs between patches and into and out of the city is ignored. Parameters used in the model are summarized in Table 1 and described in detail in the following section.

**Table 1:** Arequipa canine rabies model parameterization.

Parameter	Definition	Value [units]	Source
*ν*	Pulsed vaccination rate	Varies by year, *microred*, and strategy	S4 Text
*α*	Death rate attributable to rabies	Varies by *microred* due to heterogeneous focus control efforts	S2 Text
**c**	Contact matrix	Normalized inverse of Euclidean distance between each *microred* pair	S3 Text
*θ*	Birth rate	10N (1−N/N_0_) [1/days], where N is the population size of the patch and N_0_ is the initial population size	Simulated to maintain equilibrium
*μ*	Background death rate	1/1099.2 [1/days]	[11]
*γ*	Incubation rate	1/22.3 [1/days]	[6]
*β*	Within-patch transmission coefficient	0.00001	“Transmission coefficient” methods below
$\beta_{c}$	Between-patch transmission coefficient	0.00001	“Transmission coefficient” methods below

#### Total population size.

We considered the *microred* (subdivision of district) to be the spatial level of patches for the Arequipa rabies metapopulation model due to the *microred* being the level at which control strategies are planned and implemented in Arequipa (Fig 3). We used dog population estimates published by the Arequipa Ministry of Health [22]. The model was seeded with 100 infectious dogs at time 0.

#### Birthrate and death rate.

Background death rate (deaths not attributable to rabies) of dogs from all disease state compartments was estimated to be 1/1099.2 days from demographic dog data from Arequipa [13]. The birthrate was selected based on simulation to keep the population numbers in equilibrium.

#### Incubation period.

The incubation period for canine rabies is well characterized in the rabies literature. We used estimates reported from a contact tracing study conducted by Hampson et al, in Tanzania that describes a gamma distribution of the incubation period with a mean of 22.3 days [8].

#### Infectious period.

**Fig 4:**
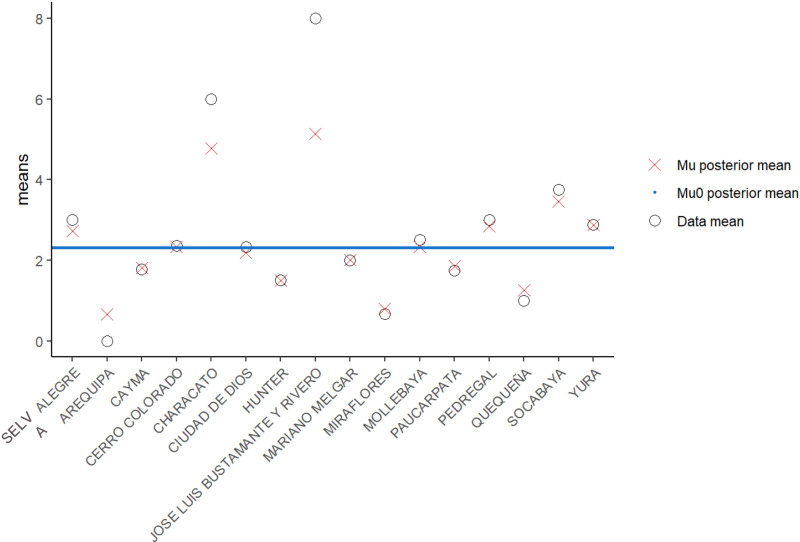
Representation of posterior mean adjustment for infectious period by district in Arequipa from hierarchical model.

Since 2015, we have collected epidemiologic data about rabid dog cases. Data include location at the district level, the date of initial signs, and the date of the dogs’ death. We have data collected on every verified rabies case from 2015 to October 2022 (n = 266) with complete date information available for 206 cases. The death rate of dogs attributable to rabies potentially varies between microreds due to different microred-level public health policies leading to heterogenous response times to rabid dog reports. We constructed a Bayesian hierarchical model to estimate the mean infectious period length in all districts (Fig 4). Briefly, we allowed information to be shared on infectious periods between districts, shrinking each individual district estimate towards the city mean. Priors of hyperparameters were non-informative with posteriors of both the city mean and the district means assumed to be gamma distributed. Complete details of infectious period parameterization and code are available in the supplement (S2 Text).

#### Transmission coefficients.

The model was simplified to incorporate only two transmission coefficients. The within-patch transmission coefficient was assumed to be constant for all patches (*microreds*). The between-patch transmission coefficient was assumed to be a constant scaled proportional by distance (km) between pairs of *microreds*. A contact matrix, **c**, was created by finding the normalized inverse Euclidean distance between the centroid of each pair of *microreds*. See supplemental information for complete methods and calculated distances between pairs (S3 Text). The between-patch transmission coefficient was then assumed to be a scalar multiplier, and applied to the inverse distance matrix to create the final contact matrix between *microreds*.

**Fig 5:**
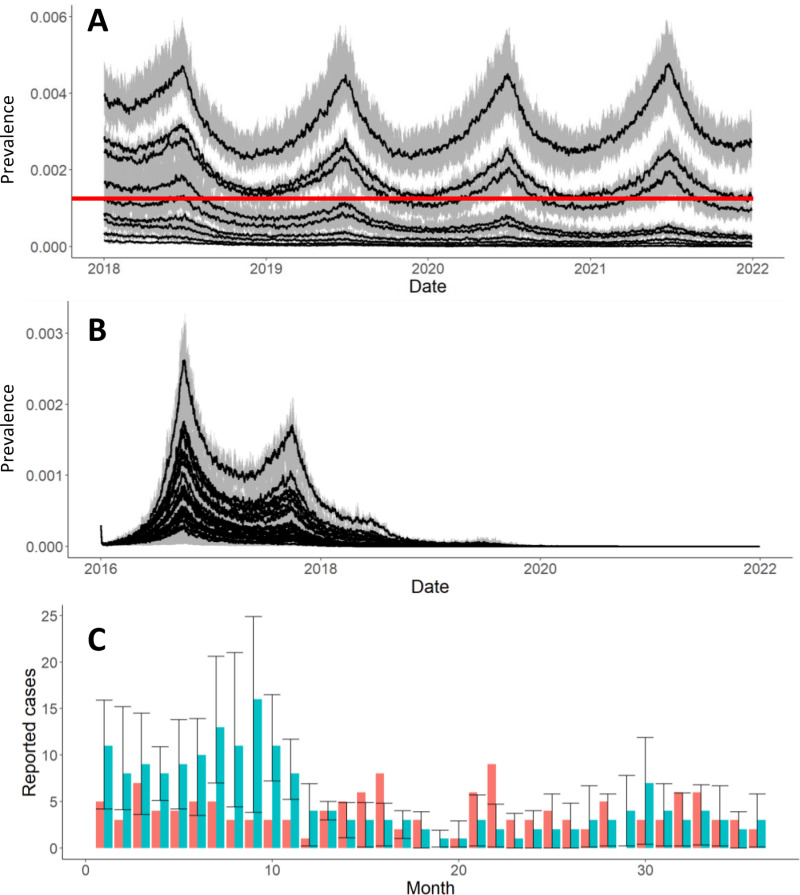
Transmission coefficient fitting. The joint parameter space of the within-patch and between-patch transmission coefficient was limited so that rabies dynamics remained persistent but at a low prevalence (<0.15%, red line) with suboptimal (50% coverage) vaccination (A), yet is eliminated successfully at adequate (70%) vaccination (B). Within the range of possible transmission coefficients, the simulations with a sampled 10% detection rate (blue bars with 95% sampling interval) were selected as the best fit via least squares to reported aggregate monthly cases (pink bars) of all *microreds* (C).

As transmission coefficients are difficult to estimate from field data, we estimated these parameters jointly through repeated simulation. Parameter ranges were restricted to match dynamics observed in the field and globally; chiefly that rabies virus persists at very low prevalence levels (<0.15%) [27] at sub-optimal vaccination coverage (50%) as seen in the field in Arequipa and globally (Fig 5A), yet the WHO recommended coverage of 70% leads to local elimination (Fig 5B) [5]. Within this range of transmission coefficients the best fit of the mean of 10 simulations compared to aggregated monthly rabies case reports, assuming a detection probability of 10%, distributed over the *microreds* based on the proportion of samples submitted [13] was used to select transmission coefficients (Fig 5C). Ranges of the within and between-patch transmission coefficient were explored jointly examined over a 0.000001 step interval from 0 to 0.0002. The within-patch transmission coefficient was estimated to be 0.00001 and the scalar between-patch transmission coefficient was estimated to be 0.00001.

#### Vaccination rate.

Vaccination rate was pulsed in the model as a time-varying parameter to simulate vaccination campaigns where many dogs are vaccinated simultaneously. We tested scenarios where all patches were vaccinated at once (i.e., the gold standard ideal recommended by PAHO [28]) and scenarios where the vaccination campaign was spread out over several months (i.e., the 2022 mass dog vaccination campaign in Arequipa). In each of these two vaccination schemes, we tested both ideal coverage (80%) and insufficient coverage (50%) levels to explore different possible scenarios. In the staggered campaign scenarios, pulses were timed and ordered according to the planned 2022 vaccination campaign, where each weekend a different *microred* was scheduled to have a *microred* level mass vaccination campaign.

#### Forward simulation.

To simulate different vaccination strategies in Arequipa, Peru, 100 simulations were run for 6 years with the parameters described in Table 1, investigating 4 different vaccination scenarios: 1) single pulse vaccination campaign reaching a sub-optimal vaccination coverage of 50%, 2) single pulse vaccination campaign reaching a policy-based vaccination coverage of 80%, 3) staggered campaign over 6 months (following 2022 plan) reaching 50% coverage, 4) staggered campaign over 6 months period reaching 80% coverage. The 50% sub-optimal vaccination coverage simulation was selected because it is below the 70% minimum coverage estimated to prevent rabies epidemics [2] and is in line with coverage achieved in Arequipa before the COVID pandemic [13]. The 80% policy-based coverage simulation was selected because it is the level recommended for Peru by the Pan-American Health Organization [28].

### Computation and mapping

All computations and figures were done using R [29]. Code and data are available on a public GitHub repository: https://github.com/bhraynor/RabiesPatchVax. The background layer of maps is from OpenStreetMap [30], and maps were plotted using the leaflet R package [25]. The *microreds* boundary data was collected by members of the ZDRC in conjunction with the Ministry of Health.

## Results

### Theoretical model sample simulations

**Fig 6:**
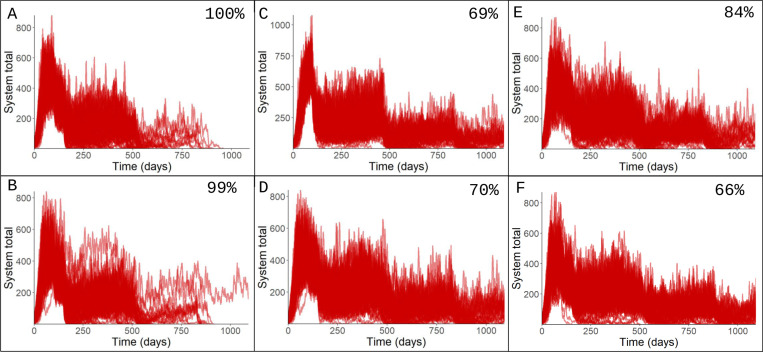
Infectious daily prevalence depicted for hypothetical model run under different scenarios of staggered, pulsed vaccination. 100 stochastic iterations were run of each scenario. Depicted on each output is the proportion of stochastic iterations where the disease was eliminated from the system. The scenarios represented here are A) Fully-connected system, all patches reaching high vaccination coverage, B) Semi-connected system, all patches reaching high vaccination coverage; C) Fully-connected system, second patch suboptimally vaccinated; D) Semi-connected system, middle patch suboptimally vaccinated second; E) Semi-connected system, edge patch suboptimally vaccinated second; F) semi-connected system, edge patch suboptimally vaccinated last.

To demonstrate the system’s dynamics and highlight the effect of different control strategies, we ran a series of simulations of a hypothetical disease outbreak on two simplified metapopulation structures (Fig 2), a 3-patch fully-connected system and a 3-path semi-connected system. All of the simulations are available in the supplement (S1 Text). Depicted in Fig 6 are the effects of metapopulation dynamics on simple control strategies that we consider later with canine rabies: instant pulse where all ‘patches’ are vaccinated on the same day and staggered pulses where different patches are vaccinated at different times.

We found that for this set of parameters, given a high level of vaccination in all patches, the proportion of stochastic iterations that reached system elimination was 99–100%, in both the fully-connected and the semi-connected systems. Conversely, if one patch in the system was suboptimally vaccinated, the proportion of stochastic iterations that reached system elimination was substantially decreased, ranging from 69%–80% for the fully-connected system depending on the order of vaccination of the patches. Similarly, for the semi-connected system the proportion of stochastic iterations achieving elimination ranged from 64% to 84%. For the semi-connected system, higher chances of elimination (84%) were achieved when the most connected (central) patch was highly vaccinated, and the worst proportions achieved when the sub-optimally vaccinated patch was ordered last. These results suggest that the timing of a vaccination strategy can influence strategy efficacy even when the vaccination coverage is the same.

### Arequipa rabies simulations

Simulations of the mass dog vaccination campaign in Arequipa were run exploring different vaccination strategies. All code and simulation output are available in the supplement (S4 Text). Overall, we found that reaching optimal vaccination coverage was the most important factor in eliminating canine rabies. In strategies where the policy-recommended level of vaccination coverage (80%) was reached, canine rabies was eliminated from the city, and in strategies where only a sub-optimal vaccination coverage (50%) was reached, canine rabies persisted to the end of the simulated 6 year time period. However, the time to elimination and total number of infected dogs increased with the staggered vaccination campaign strategies.

**Fig 7:**
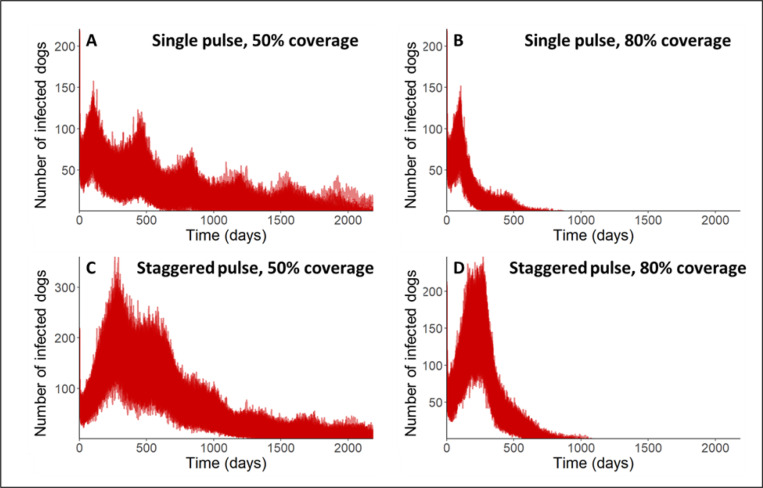
City-wide simulation scenario results. The aggregated simulated citywide rabies incidence is plotted in red. The different strategies tested were a single pulse campaign reaching 50% coverage (A) and 80% coverage (B) as well as a 6-month staggered campaign reaching 50% converge (C) and 80% coverage (D).

When looking at the dynamics of the city dog population (the sum of all *microreds*), overall trends of the different control strategies can be examined (Fig 7 and Table 2). Individual patch dynamics are depicted in the supplement (S4 Text). At 50% vaccination coverage levels, both strategies (single pulse and staggered pulse) failed to achieve canine rabies elimination in 100% of stochastic iteration (Fig 7A and 7C), though the single-pulse elimination got close (97%). At 80% coverage, both strategies led to elimination in every stochastic iteration (Fig 7B and 7D). The single pulse 80% coverage vaccination strategy had an average of 258 days shorter mean time to elimination compared to the staggered vaccination strategy. In terms of epidemic size, the average total number of cases over the 6-year simulations coverage was more than 5× times more in the staggered strategy compared to the single pulse at 80% coverage and more than 6× more at 50% coverage (Table 2). Our results are based on a system with variable survival times of rabid dogs based on microred-level policy. These results substantially change if survival times are homogeneous. In a sensitivity analysis looking at homogenous death rates, all stochastic iterations in all scenarios, including suboptimal vaccination scenarios, resulted in elimination within 6 years with increased time to elimination in all cases (S5 Text). These findings highlight how heterogeneous population dynamics can complicate elimination prospects.

**Table 2:** Arequipa City rabies control strategy scenario summary results.

Strategy	Proportion of iterations achieving elimination	Days to elimination, if reached (95% sampling interval)	6-year infected count, (95% sampling interval)
Single pulse, 50% coverage	0.97	1520 (1041, 2126)	4897 (2675, 8470)
Single pulse, 80% coverage	1.0	567 (426, 716)	1812 (1052, 2520)
Staggered, 50% coverage	0.72	1952 (1546, 2188)	26850 (15582,40074)
Staggered, 80% coverage	1.0	826 (711, 966)	9,324 (6051, 13168)

## Discussion

In our model simulations, we found that a staggered vaccination campaign can theoretically eliminate canine rabies from Arequipa, though with an elongated timeframe of several months. Due to feasibility constraints, there may be tradeoffs between reaching a sufficient coverage level and conducting a citywide vaccination campaign in one weekend. Our findings suggest that staggered-pulse rabies mass dog vaccination campaigns are a potential solution when single-pulse vaccination campaigns reaching adequate coverage are infeasible. Single-pulse vaccination campaigns outperform staggered-pulse vaccination campaigns for equivalent coverage in terms of cases averted and time-to-elimination. However, staggered-pulse vaccination campaigns can achieve regional elimination if real-world constraints make them the most feasible option to reach sufficient coverage. Additionally, our simulations predict a great discrepancy between single- and staggered-pulse campaigns with single-pulse campaigns having between 18–20% of the number of cases over the 6-year simulation period as the equivalent staggered-pulse campaign in part due to higher amplitudes of peaks and in part due to increased time to elimination. This increased case load adds a greater public health burden as well as increased opportunity for persistence, especially if vaccination campaigns are disrupted from year-to-year as occurred notably during the COVID-19 pandemic [13]. Ultimately, achieving sustained yearly high vaccination coverage is crucial for elimination prospects. While single-weekend campaigns reaching equivalent vaccination coverage as staggered campaigns may achieve elimination faster (within a few months), planners should carefully assess feasibility and anticipated coverage when scheduling mass dog vaccination campaigns.

There are various barriers to a mass vaccination campaign on the scale of approximately 175,000 vaccinations (to reach 70% coverage in Arequipa); including personnel and operation management on such a scale. In the past, Arequipa City mass dog vaccination campaigns have consistently failed to reach sufficient coverage [13,31,32]. From 2016–2020, campaigns reached an average of approximately 54% with the COVID-19 pandemic further disrupting vaccination campaign efforts [13]. In an attempt to reach adequate coverage, a staggered campaign was proposed for 2022. However, a staggered campaign does present new challenges to a cohesive campaign. One of the largest barriers to participating in the mass dog vaccination campaign is knowledge of the campaign through effective communication [31,32]. Globally, rabies mass dog vaccination campaigns are often coordinated around World Rabies Day (September 28), and partnered with rabies awareness events and educational drives [31,33]. A staggered campaign can lose the cohesive communication messaging and add confusion, especially for people who live near the borders of their *microreds*.

This model has several limitations. Several simplifying assumptions were made chiefly, birth rate, death rate, and transmission coefficients were assumed to be constant in all patches in our system although, in reality, they likely vary. The between-patch transmission coefficients were assumed to be proportional to distance; however, there are other geographical features like roads and urban corridors that may act as facilitators or barriers to dog movement [34]. We focused on the policies that are made at the *microred* level including rabies control and vaccination campaign strategies. Within patches, populations were assumed to be homogenous and completely mixed. We considered only the owned dog population - these are the dogs characterized by the Ministry of Health and those that would be able to participate in the mass dog vaccination campaigns. In Arequipa, many dogs are free-roaming. Most of the dogs have owners but are allowed various access to the street. However, it is possible that within the free-roaming dog population (street dogs) are a substantial number of stray (unowned) dogs that are uncharacterized and not considered in the model. Finally, rabies is massively underreported globally [5,35,36]. We assumed a 10% detection rate distributed across *microreds* proportionally to their submission rates in line with expert opinion from the field and published reports [13].

Spatial aspects of virus transmission are essential to understand rabies virus dynamics [37–46]. Spatial transmission patterns are thought to drive the low-level persistence of the virus. One proposed mechanism of virus low-level persistence in dogs is local depletion of susceptibles where clusters of dogs around a case get re-exposed multiple times [27]. Interestingly, in Arequipa, the dog population is very sizable throughout the city, and dog movements throughout the city make the susceptible pool practically infinite [34], yet similar low-prevalence persistent rabies virus dynamics are observed. We introduce a meta-population model that elucidates dynamics by incorporating control policy-induced heterogeneity within the dog population.

Our findings corroborate empirical evidence from Bali. Townsend et al present a model of a staggered pulse vaccination campaign in Bali, Indonesia where secondary rabies cases through time were allocated in a spatially explicit representative grid of Bali, and different vaccination coverages per each grid cell were simulated under different strategies. They find that the probability of canine elimination is roughly equivalent in campaigns spread out over 1 month versus those spread out over 6 months [10]. Notably, in the past 10 years, Bali has successfully controlled canine rabies using staggered mass vaccination campaigns reaching 70% coverage in combination with other strategies [47].

Our metapopulation model accounts for *microred-*level differences in policies. Since 2015, Arequipa has reached sub-optimal and often patchy coverage in the annual mass dog vaccination campaign. Metapopulation dynamics account for the continued persistence of rabies in the Arequipa free-roaming dog population. Our hypothetical model found that the underlying connectivity structure of the population had an impact on disease outcomes but underlined the importance of adequate vaccination. In relation to general metapopulation models with pulsed vaccinations and more specifically to rabies in Arequipa, open questions remain about how the order of vaccinations impacts prospects of elimination. Additionally, strategies that target more connected regions could be explored. More research is needed to understand the underlying dog ecology in free-roaming dogs in Arequipa and other rabies-endemic regions. Our metapopulation model results question the current paradigm in rabies control that the only way to eliminate rabies is through yearly mass vaccination campaigns conducted simultaneously across the region. Our model suggests that a staggered campaign can lead to rabies elimination with the tradeoffs of increased time and size of the epidemic. This leaves room for economic models to optimize these tradeoffs of feasibility and epidemiologic outcomes to analyze the ideal strategy for specific scenarios. However, in light of continued canine rabies persistence in places like Arequipa, we propose exploring strategies beyond the current paradigm in order to problem-solve and adapt to change.

## Conclusions

We present a model that accounts for local variability in control measure policy and population dynamics to better inform policymakers in creating strategies for local rabies elimination. The current paradigm of a single-weekend or single-day mass dog vaccination campaign to eliminate canine rabies has historically been effective, so other campaign timing strategies are not considered. While single-pulse vaccination strategies have proven to be effective, they pose logistical challenges, particularly in areas with large dog populations and low levels of funding. We found that a staggered mass dog vaccination campaign could be effective in eliminating rabies when feasibility constraints make reaching adequate coverage in a single weekend campaign impossible. Our results suggest that a shifting paradigm can be advantageous to both local policy-makers and the worldwide goal of ‘zero human deaths by 2030’. The staggered mass dog vaccination campaign was implemented in Arequipa City in 2022, with plans to improve implementations in the following years.

## Supporting information

S1 Text Supplementary R Markdown HTML output of theoretical model simulations. HTML file can be opened in any web browser.
(HTML)

S2 Text Supplementary R Markdown HTML output of Bayesian hierarchical model. HTML file can be opened in any web browser.
(HTML)

S3 Text Supplementary R Markdown HTML output of parameterization methods. Base map and data from OpenStreetMap and OpenStreetMap Foundation (26,27). HTML file can be opened in any web browser.
(HTML)

S4 Text Supplementary R Markdown HTML output of vaccination strategy simulations. HTML file can be opened in any web browser.
(HTML)

S5 Text Supplementary R Markdown HTML output of sensitivity analysis with homogenous rabid dog survival times. HTML file can be opened in any web browser.
(HTML)
