# Supplementary material for: Challenging a paradigm: Staggered versus single-pulse mass dog vaccination strategy for rabies elimination
Source: PLoS Comput Biol. 2025 Feb 7;21(2):e1012780. doi: 10.1371/journal.pcbi.1012780 (PMC11805426; doi:10.1371/journal.pcbi.1012780)
Supplement: S1 Text — HTML file can be opened in any web browser. (HTML) [file pcbi.1012780.s001.html]

Simulations\_ToyModel


# Simulations\_ToyModel

#### B. Raynor

#### 3/27/2023

## Set up

Load packages, functions, data

```
#clear environment
rm(list = ls()) 

#load required packages
library(ggplot2)  #pretty figures
library(dplyr)    #tidy code
library(ggpubr)   #plot arrangement

source(here::here("R", "Functions_StochasticModels.R"))
source(here::here("R", "Functions_StochasticSimulationVisualization.R"))

#parameters
parms <- data.frame( 
  patch = c("Patch 1", "Patch 2", "Patch 3"),
  b= c(10,10,10),
  beta = c(0.001, 0.001, 0.001),
  mu = c(1/5000, 1/5000, 1/5000),
  alpha = c(1/3, 1/3, 1/3),
  nu1 = c(0,0,0),
  gamma = c(1/2, 1/2, 1/2),
  N = c(1000, 1000, 1000),
  current_infect = c(1,1,1))

knitr::kable(parms %>% select(-current_infect))
```

| patch | b | beta | mu | alpha | nu1 | gamma | N |
| --- | --- | --- | --- | --- | --- | --- | --- |
| Patch 1 | 10 | 0.001 | 2e-04 | 0.3333333 | 0 | 0.5 | 1000 |
| Patch 2 | 10 | 0.001 | 2e-04 | 0.3333333 | 0 | 0.5 | 1000 |
| Patch 3 | 10 | 0.001 | 2e-04 | 0.3333333 | 0 | 0.5 | 1000 |

**Table:** Synthetic parameters used to explore general
SEIV model

Initialize SDEs

```
num_patch = length(parms$patch)

#initial conditions 
init <- data.frame(S=0.5*parms$N - 10*parms$current_infect,
                   E= rep(0,num_patch),
                   I= 10*parms$current_infect,
                   V= 0.5*parms$N)

#time series
num_years = 3
Time = 365*num_years #End time
dt = 1 #Step size dt

#How many simulations we want to average
sim=100

#pulse vax
vax0= matrix(0, Time/dt, num_patch)
```

## PART A: 3 patches all connected

```
#contact matrix
df.contact <-data.frame(patch1 = c(0,1,1), patch2= c(1,0,1), patch3 =c(1,1,0))
beta.contact_constant = 0.00001 
beta.contact = rfun.FormatContact(df.contact, beta.contact_constant)
```

Sim 1: Single pulse, all vaccinated

```
set.seed(123)

#set up VANCAN pulse schedules
vax <- vax0
vax[c(100, (100+365), (100+2*365)),] = c(rep(0.7, num_patch)) #

#save simulations together in a big df
out <- NULL
for(i in 1:sim){
  sub <-  rfun.PatchSEIV(init, parms, beta.contact, vax, Time, dt)%>%
    mutate(sim = i)
  out <- rbind(out, sub)
}

#assess time to elimination

df.system_elimination <- data.frame(avg_time_elimination = NULL, percent_elim = NULL)


rfun_EliminationTime <- function(out){
  df <- out %>% 
    select(starts_with("I"), time, sim)%>% 
    tidyr::pivot_longer(cols=!c(time, sim), names_to="patch")%>%
    filter(value != 0)%>%
    group_by(sim, patch)%>%
    summarize(last_case= max(time))%>%
    ungroup()%>%
    mutate(elim_patch = ifelse(last_case == 1095, 1, 0))
  
  df.elim <- df %>% #only for patches that do get eliminated
    group_by(sim)%>%
    summarise(elim_system = sum(elim_patch))%>%
    filter(elim_system == 0)
  
  num_elim = nrow(df.elim)
    
  
  df <- df %>%
    filter(elim_patch == 0)%>%
    group_by(patch)%>%
    summarise(mean = round(mean(last_case),0),
              SI_2.5 = round(quantile(last_case, .025),0),
              SI_97.5 = round(quantile(last_case, .975),0),
              num_sim=n())
  
  df.system_elimination <<- rbind(df.system_elimination, c(max(df$mean), num_elim/sim))

  
  return(knitr::kable(df))
}

rfun_RabiesDynamics(out, parms$patch)
rfun_EliminationTime(out)
```

| patch | mean | SI\_2.5 | SI\_97.5 | num\_sim |
| --- | --- | --- | --- | --- |
| I1 | 437 | 128 | 845 | 100 |
| I2 | 448 | 126 | 851 | 100 |
| I3 | 446 | 128 | 838 | 100 |

Sim 2: Staggered pulse, all vaccinated (patch 1 –> patch 2 –>
patch 3)

```
set.seed(123)

#set up VANCAN pulse schedules
vax <- vax0
vax[c(50, (50+365), (50+2*365)), 1] = c(rep(0.7, num_patch)) #
vax[c(100, (100+365), (100+2*365)), 2] = c(rep(0.7, num_patch)) #
vax[c(150, (150+365), (150+2*365)), 3] = c(rep(0.7, num_patch)) #


#save simulations together in a big df
out <- NULL
for(i in 1:sim){
  sub <-  rfun.PatchSEIV(init, parms, beta.contact, vax, Time, dt)%>%
    mutate(sim = i)
  out <- rbind(out, sub)
}

rfun_RabiesDynamics(out, parms$patch)
rfun_EliminationTime(out)
```

| patch | mean | SI\_2.5 | SI\_97.5 | num\_sim |
| --- | --- | --- | --- | --- |
| I1 | 458 | 153 | 843 | 100 |
| I2 | 463 | 127 | 877 | 100 |
| I3 | 482 | 173 | 845 | 100 |

Sim 3: Staggered pulse, all vaccinated (patch 2 –> patch 1 –>
patch 3)

```
set.seed(123)

#set up VANCAN pulse schedules
vax <- vax0
vax[c(50, (50+365), (50+2*365)), 2] = c(rep(0.7, num_patch)) #
vax[c(100, (100+365), (100+2*365)), 1] = c(rep(0.7, num_patch)) #
vax[c(150, (150+365), (150+2*365)), 3] = c(rep(0.7, num_patch)) #


#save simulations together in a big df
out <- NULL
for(i in 1:sim){
  sub <-  rfun.PatchSEIV(init, parms, beta.contact, vax, Time, dt)%>%
    mutate(sim = i)
  out <- rbind(out, sub)
}

rfun_RabiesDynamics(out, parms$patch)
rfun_EliminationTime(out)
```

| patch | mean | SI\_2.5 | SI\_97.5 | num\_sim |
| --- | --- | --- | --- | --- |
| I1 | 421 | 118 | 872 | 100 |
| I2 | 425 | 132 | 834 | 100 |
| I3 | 454 | 170 | 860 | 100 |

Sim 4: Instant pulse, 2 vaccinated (70%), 1 (patch 1) vaccinated
(30%)

```
set.seed(123)

#set up VANCAN pulse schedules
vax <- vax0
vax[c(100, (100+365), (100+2*365)), 1] = c(rep(0.3, num_patch)) #
vax[c(100, (100+365), (100+2*365)), 2] = c(rep(0.7, num_patch)) #
vax[c(100, (100+365), (100+2*365)), 3] = c(rep(0.7, num_patch)) #


#save simulations together in a big df
out <- NULL
for(i in 1:sim){
  sub <-  rfun.PatchSEIV(init, parms, beta.contact, vax, Time, dt)%>%
    mutate(sim = i)
  out <- rbind(out, sub)
}

rfun_RabiesDynamics(out, parms$patch)
rfun_EliminationTime(out)
```

| patch | mean | SI\_2.5 | SI\_97.5 | num\_sim |
| --- | --- | --- | --- | --- |
| I1 | 669 | 227 | 1033 | 73 |
| I2 | 681 | 190 | 1092 | 91 |
| I3 | 683 | 251 | 1091 | 91 |

Sim 5: Instant pulse, 2 vaccinated (70%), 1 (patch 2) vaccinated
(30%)

```
set.seed(123)

#set up VANCAN pulse schedules
vax <- vax0
vax[c(100, (100+365), (100+2*365)), 1] = c(rep(0.7, num_patch)) #
vax[c(100, (100+365), (100+2*365)), 2] = c(rep(0.3, num_patch)) #
vax[c(100, (100+365), (100+2*365)), 3] = c(rep(0.7, num_patch)) #


#save simulations together in a big df
out <- NULL
for(i in 1:sim){
  sub <-  rfun.PatchSEIV(init, parms, beta.contact, vax, Time, dt)%>%
    mutate(sim = i)
  out <- rbind(out, sub)
}

rfun_RabiesDynamics(out, parms$patch)
rfun_EliminationTime(out)
```

| patch | mean | SI\_2.5 | SI\_97.5 | num\_sim |
| --- | --- | --- | --- | --- |
| I1 | 694 | 255 | 1088 | 94 |
| I2 | 688 | 316 | 1020 | 73 |
| I3 | 708 | 321 | 1093 | 95 |

Sim 6: Staggered pulse, all vaccinated (patch 1 (70%) –> patch 2
(30%) –> patch 3 (70%))

```
set.seed(123)

#set up VANCAN pulse schedules
vax <- vax0
vax[c(50, (50+365), (50+2*365)), 1] = c(rep(0.7, num_patch)) #
vax[c(100, (100+365), (100+2*365)), 2] = c(rep(0.3, num_patch)) #
vax[c(150, (150+365), (150+2*365)), 3] = c(rep(0.7, num_patch)) #


#save simulations together in a big df
out <- NULL
for(i in 1:sim){
  sub <-  rfun.PatchSEIV(init, parms, beta.contact, vax, Time, dt)%>%
    mutate(sim = i)
  out <- rbind(out, sub)
}

rfun_RabiesDynamics(out, parms$patch)
rfun_EliminationTime(out)
```

| patch | mean | SI\_2.5 | SI\_97.5 | num\_sim |
| --- | --- | --- | --- | --- |
| I1 | 703 | 180 | 1091 | 92 |
| I2 | 674 | 222 | 1026 | 69 |
| I3 | 697 | 171 | 1093 | 91 |

Sim 7: Staggered pulse, all vaccinated (patch 2 (70%) –> patch 1
(30%) –> patch 3 (70%))

```
set.seed(123)

#set up VANCAN pulse schedules
vax <- vax0
vax[c(50, (50+365), (50+2*365)), 2] = c(rep(0.7, num_patch)) #
vax[c(100, (100+365), (100+2*365)), 1] = c(rep(0.3, num_patch)) #
vax[c(150, (150+365), (150+2*365)), 3] = c(rep(0.7, num_patch)) #


#save simulations together in a big df
out <- NULL
for(i in 1:sim){
  sub <-  rfun.PatchSEIV(init, parms, beta.contact, vax, Time, dt)%>%
    mutate(sim = i)
  out <- rbind(out, sub)
}

rfun_RabiesDynamics(out, parms$patch)
rfun_EliminationTime(out)
```

| patch | mean | SI\_2.5 | SI\_97.5 | num\_sim |
| --- | --- | --- | --- | --- |
| I1 | 725 | 221 | 1058 | 79 |
| I2 | 695 | 157 | 1093 | 95 |
| I3 | 721 | 185 | 1088 | 94 |

Sim 8: Staggered pulse, all vaccinated (patch 1 (30%) –> patch 2
(70%) –> patch 3 (70%))

```
set.seed(123)

#set up VANCAN pulse schedules
vax <- vax0
vax[c(50, (50+365), (50+2*365)), 1] = c(rep(0.3, num_patch)) #
vax[c(100, (100+365), (100+2*365)), 2] = c(rep(0.7, num_patch)) #
vax[c(150, (150+365), (150+2*365)), 3] = c(rep(0.7, num_patch)) #


#save simulations together in a big df
out <- NULL
for(i in 1:sim){
  sub <-  rfun.PatchSEIV(init, parms, beta.contact, vax, Time, dt)%>%
    mutate(sim = i)
  out <- rbind(out, sub)
}

rfun_RabiesDynamics(out, parms$patch)
rfun_EliminationTime(out)
```

| patch | mean | SI\_2.5 | SI\_97.5 | num\_sim |
| --- | --- | --- | --- | --- |
| I1 | 645 | 351 | 1002 | 80 |
| I2 | 654 | 187 | 1086 | 94 |
| I3 | 675 | 199 | 1090 | 96 |

Sim 9: Staggered pulse, all vaccinated (patch 2 (30%) –> patch 1
(70%) –> patch 3 (70%))

```
set.seed(123)

#set up VANCAN pulse schedules
vax <- vax0
vax[c(50, (50+365), (50+2*365)), 2] = c(rep(0.3, num_patch)) #
vax[c(100, (100+365), (100+2*365)), 1] = c(rep(0.7, num_patch)) #
vax[c(150, (150+365), (150+2*365)), 3] = c(rep(0.7, num_patch)) #


#save simulations together in a big df
out <- NULL
for(i in 1:sim){
  sub <-  rfun.PatchSEIV(init, parms, beta.contact, vax, Time, dt)%>%
    mutate(sim = i)
  out <- rbind(out, sub)
}

rfun_RabiesDynamics(out, parms$patch)
rfun_EliminationTime(out)
```

| patch | mean | SI\_2.5 | SI\_97.5 | num\_sim |
| --- | --- | --- | --- | --- |
| I1 | 661 | 187 | 1091 | 96 |
| I2 | 663 | 398 | 1015 | 78 |
| I3 | 678 | 173 | 1093 | 95 |

## PART B: 3 patches: patch 1 <-> patch 2 <-> patch 3

```
#contact matrix
df.contact <-data.frame(patch1 = c(0,1,0), patch2= c(1,0,1), patch3 =c(0,1,0))
beta.contact_constant = 0.00001 
beta.contact = rfun.FormatContact(df.contact, beta.contact_constant)
```

Sim 1: Single pulse, all vaccinated

```
set.seed(123)

#set up VANCAN pulse schedules
vax <- vax0
vax[c(100, (100+365), (100+2*365)),] = c(rep(0.7, num_patch)) #

#save simulations together in a big df
out <- NULL
for(i in 1:sim){
  sub <-  rfun.PatchSEIV(init, parms, beta.contact, vax, Time, dt)%>%
    mutate(sim = i)
  out <- rbind(out, sub)
}

rfun_RabiesDynamics(out, parms$patch)
rfun_EliminationTime(out)
```

| patch | mean | SI\_2.5 | SI\_97.5 | num\_sim |
| --- | --- | --- | --- | --- |
| I1 | 408 | 122 | 845 | 99 |
| I2 | 447 | 122 | 841 | 100 |
| I3 | 401 | 123 | 699 | 100 |

Sim 2: Staggered pulse, all vaccinated (patch 1 –> patch 2 –>
patch 3)

```
set.seed(123)

#set up VANCAN pulse schedules
vax <- vax0
vax[c(50, (50+365), (50+2*365)), 1] = c(rep(0.7, num_patch)) #
vax[c(100, (100+365), (100+2*365)), 2] = c(rep(0.7, num_patch)) #
vax[c(150, (150+365), (150+2*365)), 3] = c(rep(0.7, num_patch)) #


#save simulations together in a big df
out <- NULL
for(i in 1:sim){
  sub <-  rfun.PatchSEIV(init, parms, beta.contact, vax, Time, dt)%>%
    mutate(sim = i)
  out <- rbind(out, sub)
}

rfun_RabiesDynamics(out, parms$patch)
rfun_EliminationTime(out)
```

| patch | mean | SI\_2.5 | SI\_97.5 | num\_sim |
| --- | --- | --- | --- | --- |
| I1 | 364 | 90 | 832 | 100 |
| I2 | 454 | 154 | 873 | 99 |
| I3 | 452 | 168 | 869 | 99 |

Sim 3: Staggered pulse, all vaccinated (patch 1 –> patch 3 –>
patch 2)

```
set.seed(123)

#set up VANCAN pulse schedules
vax <- vax0
vax[c(50, (50+365), (50+2*365)), 1] = c(rep(0.7, num_patch)) #
vax[c(100, (100+365), (100+2*365)), 3] = c(rep(0.7, num_patch)) #
vax[c(150, (150+365), (150+2*365)), 2] = c(rep(0.7, num_patch)) #


#save simulations together in a big df
out <- NULL
for(i in 1:sim){
  sub <-  rfun.PatchSEIV(init, parms, beta.contact, vax, Time, dt)%>%
    mutate(sim = i)
  out <- rbind(out, sub)
}

rfun_RabiesDynamics(out, parms$patch)
rfun_EliminationTime(out)
```

| patch | mean | SI\_2.5 | SI\_97.5 | num\_sim |
| --- | --- | --- | --- | --- |
| I1 | 369 | 150 | 668 | 100 |
| I2 | 447 | 170 | 988 | 100 |
| I3 | 399 | 122 | 824 | 99 |

Sim 4: Staggered pulse, all vaccinated (patch 2 –> patch 1 –>
patch 3)

```
set.seed(123)

#set up VANCAN pulse schedules
vax <- vax0
vax[c(50, (50+365), (50+2*365)), 2] = c(rep(0.7, num_patch)) #
vax[c(100, (100+365), (100+2*365)), 1] = c(rep(0.7, num_patch)) #
vax[c(150, (150+365), (150+2*365)), 3] = c(rep(0.7, num_patch)) #


#save simulations together in a big df
out <- NULL
for(i in 1:sim){
  sub <-  rfun.PatchSEIV(init, parms, beta.contact, vax, Time, dt)%>%
    mutate(sim = i)
  out <- rbind(out, sub)
}

rfun_RabiesDynamics(out, parms$patch)
rfun_EliminationTime(out)
```

| patch | mean | SI\_2.5 | SI\_97.5 | num\_sim |
| --- | --- | --- | --- | --- |
| I1 | 346 | 116 | 817 | 100 |
| I2 | 407 | 128 | 782 | 100 |
| I3 | 404 | 164 | 845 | 100 |

Sim 5: Instant pulse, 2 vaccinated (70%), 1 (patch 1) vaccinated
(30%)

```
set.seed(123)

#set up VANCAN pulse schedules
vax <- vax0
vax[c(100, (100+365), (100+2*365)), 1] = c(rep(0.3, num_patch)) #
vax[c(100, (100+365), (100+2*365)), 2] = c(rep(0.7, num_patch)) #
vax[c(100, (100+365), (100+2*365)), 3] = c(rep(0.7, num_patch)) #


#save simulations together in a big df
out <- NULL
for(i in 1:sim){
  sub <-  rfun.PatchSEIV(init, parms, beta.contact, vax, Time, dt)%>%
    mutate(sim = i)
  out <- rbind(out, sub)
}

rfun_RabiesDynamics(out, parms$patch)
rfun_EliminationTime(out)
```

| patch | mean | SI\_2.5 | SI\_97.5 | num\_sim |
| --- | --- | --- | --- | --- |
| I1 | 733 | 209 | 1054 | 77 |
| I2 | 719 | 366 | 1087 | 92 |
| I3 | 445 | 129 | 827 | 100 |

Sim 6: Instant pulse, 2 vaccinated (70%), 1 (patch 2) vaccinated
(30%)

```
set.seed(123)

#set up VANCAN pulse schedules
vax <- vax0
vax[c(100, (100+365), (100+2*365)), 1] = c(rep(0.7, num_patch)) #
vax[c(100, (100+365), (100+2*365)), 2] = c(rep(0.3, num_patch)) #
vax[c(100, (100+365), (100+2*365)), 3] = c(rep(0.7, num_patch)) #


#save simulations together in a big df
out <- NULL
for(i in 1:sim){
  sub <-  rfun.PatchSEIV(init, parms, beta.contact, vax, Time, dt)%>%
    mutate(sim = i)
  out <- rbind(out, sub)
}

rfun_RabiesDynamics(out, parms$patch)
rfun_EliminationTime(out)
```

| patch | mean | SI\_2.5 | SI\_97.5 | num\_sim |
| --- | --- | --- | --- | --- |
| I1 | 710 | 124 | 1092 | 92 |
| I2 | 693 | 175 | 1035 | 69 |
| I3 | 715 | 190 | 1092 | 93 |

Sim 7: Staggered pulse, all vaccinated (patch 1 (70%) –> patch 2
(30%) –> patch 3 (70%))

```
set.seed(123)

#set up VANCAN pulse schedules
vax <- vax0
vax[c(50, (50+365), (50+2*365)), 1] = c(rep(0.7, num_patch)) #
vax[c(100, (100+365), (100+2*365)), 2] = c(rep(0.3, num_patch)) #
vax[c(150, (150+365), (150+2*365)), 3] = c(rep(0.7, num_patch)) #


#save simulations together in a big df
out <- NULL
for(i in 1:sim){
  sub <-  rfun.PatchSEIV(init, parms, beta.contact, vax, Time, dt)%>%
    mutate(sim = i)
  out <- rbind(out, sub)
}

rfun_RabiesDynamics(out, parms$patch)
rfun_EliminationTime(out)
```

| patch | mean | SI\_2.5 | SI\_97.5 | num\_sim |
| --- | --- | --- | --- | --- |
| I1 | 697 | 264 | 1087 | 92 |
| I2 | 718 | 306 | 1013 | 70 |
| I3 | 740 | 239 | 1092 | 93 |

Sim 8: Staggered pulse, all vaccinated (patch 2 (70%) –> patch 1
(30%) –> patch 3 (70%))

```
set.seed(123)

#set up VANCAN pulse schedules
vax <- vax0
vax[c(50, (50+365), (50+2*365)), 2] = c(rep(0.7, num_patch)) #
vax[c(100, (100+365), (100+2*365)), 1] = c(rep(0.3, num_patch)) #
vax[c(150, (150+365), (150+2*365)), 3] = c(rep(0.7, num_patch)) #


#save simulations together in a big df
out <- NULL
for(i in 1:sim){
  sub <-  rfun.PatchSEIV(init, parms, beta.contact, vax, Time, dt)%>%
    mutate(sim = i)
  out <- rbind(out, sub)
}

rfun_RabiesDynamics(out, parms$patch)
rfun_EliminationTime(out)
```

| patch | mean | SI\_2.5 | SI\_97.5 | num\_sim |
| --- | --- | --- | --- | --- |
| I1 | 639 | 160 | 1014 | 84 |
| I2 | 661 | 162 | 1085 | 97 |
| I3 | 433 | 167 | 894 | 100 |

Sim 9: Staggered pulse, all vaccinated (patch 1 (30%) –> patch 2
(70%) –> patch 3 (70%))

```
set.seed(123)

#set up VANCAN pulse schedules
vax <- vax0
vax[c(50, (50+365), (50+2*365)), 1] = c(rep(0.3, num_patch)) #
vax[c(100, (100+365), (100+2*365)), 2] = c(rep(0.7, num_patch)) #
vax[c(150, (150+365), (150+2*365)), 3] = c(rep(0.7, num_patch)) #


#save simulations together in a big df
out <- NULL
for(i in 1:sim){
  sub <-  rfun.PatchSEIV(init, parms, beta.contact, vax, Time, dt)%>%
    mutate(sim = i)
  out <- rbind(out, sub)
}

rfun_RabiesDynamics(out, parms$patch)
rfun_EliminationTime(out)
```

| patch | mean | SI\_2.5 | SI\_97.5 | num\_sim |
| --- | --- | --- | --- | --- |
| I1 | 634 | 167 | 980 | 84 |
| I2 | 635 | 140 | 1088 | 95 |
| I3 | 460 | 169 | 841 | 100 |

Sim 10: Staggered pulse, all vaccinated (patch 2 (30%) –> patch 1
(70%) –> patch 3 (70%))

```
set.seed(123)

#set up VANCAN pulse schedules
vax <- vax0
vax[c(50, (50+365), (50+2*365)), 2] = c(rep(0.3, num_patch)) #
vax[c(100, (100+365), (100+2*365)), 1] = c(rep(0.7, num_patch)) #
vax[c(150, (150+365), (150+2*365)), 3] = c(rep(0.7, num_patch)) #


#save simulations together in a big df
out <- NULL
for(i in 1:sim){
  sub <-  rfun.PatchSEIV(init, parms, beta.contact, vax, Time, dt)%>%
    mutate(sim = i)
  out <- rbind(out, sub)
}

rfun_RabiesDynamics(out, parms$patch)
rfun_EliminationTime(out)
```

| patch | mean | SI\_2.5 | SI\_97.5 | num\_sim |
| --- | --- | --- | --- | --- |
| I1 | 648 | 233 | 1089 | 94 |
| I2 | 664 | 327 | 979 | 78 |
| I3 | 638 | 172 | 1090 | 94 |

Sim 11: Staggered pulse, all vaccinated (patch 1 (70%) –> patch 2
(70%) –> patch 3 (30%))

```
set.seed(123)

#set up VANCAN pulse schedules
vax <- vax0
vax[c(50, (50+365), (50+2*365)), 1] = c(rep(0.7, num_patch)) #
vax[c(100, (100+365), (100+2*365)), 2] = c(rep(0.7, num_patch)) #
vax[c(150, (150+365), (150+2*365)), 3] = c(rep(0.3, num_patch)) #


#save simulations together in a big df
out <- NULL
for(i in 1:sim){
  sub <-  rfun.PatchSEIV(init, parms, beta.contact, vax, Time, dt)%>%
    mutate(sim = i)
  out <- rbind(out, sub)
}

rfun_RabiesDynamics(out, parms$patch)
rfun_EliminationTime(out)
```

| patch | mean | SI\_2.5 | SI\_97.5 | num\_sim |
| --- | --- | --- | --- | --- |
| I1 | 397 | 90 | 842 | 100 |
| I2 | 777 | 222 | 1093 | 91 |
| I3 | 756 | 247 | 1059 | 64 |

Sim 12: Staggered pulse, all vaccinated (patch 2 (70%) –> patch 1
(70%) –> patch 3 (30%))

```
set.seed(123)

#set up VANCAN pulse schedules
vax <- vax0
vax[c(50, (50+365), (50+2*365)), 2] = c(rep(0.7, num_patch)) #
vax[c(100, (100+365), (100+2*365)), 1] = c(rep(0.7, num_patch)) #
vax[c(150, (150+365), (150+2*365)), 3] = c(rep(0.3, num_patch)) #


#save simulations together in a big df
out <- NULL
for(i in 1:sim){
  sub <-  rfun.PatchSEIV(init, parms, beta.contact, vax, Time, dt)%>%
    mutate(sim = i)
  out <- rbind(out, sub)
}

rfun_RabiesDynamics(out, parms$patch)
rfun_EliminationTime(out)
```

| patch | mean | SI\_2.5 | SI\_97.5 | num\_sim |
| --- | --- | --- | --- | --- |
| I1 | 373 | 116 | 887 | 100 |
| I2 | 706 | 145 | 1093 | 92 |
| I3 | 681 | 144 | 1066 | 66 |

Compare systems

```
colnames(df.system_elimination) = c("Avg_time_elim", "Percent_elim")

df.sys <- df.system_elimination %>%
  mutate(Scenario = seq(1:length(Percent_elim)))%>%
  select(Scenario, Percent_elim, Avg_time_elim)


knitr::kable(df.sys)
```

| Scenario | Percent\_elim | Avg\_time\_elim |
| --- | --- | --- |
| 1 | 1.00 | 448 |
| 2 | 1.00 | 482 |
| 3 | 1.00 | 454 |
| 4 | 0.73 | 683 |
| 5 | 0.73 | 708 |
| 6 | 0.69 | 703 |
| 7 | 0.79 | 725 |
| 8 | 0.80 | 675 |
| 9 | 0.78 | 678 |
| 10 | 0.99 | 447 |
| 11 | 0.99 | 454 |
| 12 | 0.99 | 447 |
| 13 | 1.00 | 407 |
| 14 | 0.77 | 733 |
| 15 | 0.69 | 715 |
| 16 | 0.70 | 740 |
| 17 | 0.84 | 661 |
| 18 | 0.84 | 635 |
| 19 | 0.78 | 664 |
| 20 | 0.64 | 777 |
| 21 | 0.66 | 706 |
