# Supplementary material for: Challenging a paradigm: Staggered versus single-pulse mass dog vaccination strategy for rabies elimination
Source: PLoS Comput Biol. 2025 Feb 7;21(2):e1012780. doi: 10.1371/journal.pcbi.1012780 (PMC11805426; doi:10.1371/journal.pcbi.1012780)
Supplement: S2 Text — HTML file can be opened in any web browser. (HTML) [file pcbi.1012780.s002.html]

Bayes Hierarchical Model


# Bayes Hierarchical Model

#### Brinkley Raynor

#### 4/29/2022

## Data

The data here is extracted dates from focus control reports. These
are the reports ascertaining the epidemiologic history of positive
canine rabies cases.For the purposes of this analysis, infectious period
(CullLag) is defined as the time between initial signs onset and
death.

```
#clean environment
rm(list = ls())

#call packages
library(dplyr) #tidy coding
library(ggplot2) #pretty figures

#Read in data
df.fc<- readRDS(here::here("data_minimal", "DeathTime.Rda"))
```

            
**Figure 1: Boxplot visualization of infectious period (dog
euthanasia date - initial rabies signs date) distribution by
district**

## Model formulation

Observations \(y\_ij\) were modeled
as a hierarchical model where there are \(i\) observations in each \(j\) district of \(y\) observed days taken to euthanize
detected rabid dogs. The district means, \(mu\_j\) are modeled as gamma distributed
with information sharing between districts.

### Gibbs Sampler

```
set.seed(123)
#GIBBS SAMPLER FUNCTION
fun.gibbs <- function(numsamp, sigsq, tausq, mu0, df){
  df.fc <- df
  
  #Format data 
  df.district = df.fc %>% dplyr::select(id, District, CullLag)%>%
    group_by(District) %>%
    summarise(mean = mean(CullLag, na.rm=TRUE), n = n())
  
  #Calculate statistics
  means=df.district$mean
  n=df.district$n
  m <- length(means)
  ntot <- sum(n)
  
  #Set up frames to hold data
  sigsq.samp <- rep(NA,numsamp)
  tausq.samp <- rep(NA,numsamp)
  mu0.samp <- rep(NA,numsamp)
  mu.samp <- matrix(NA,nrow=numsamp,ncol=m)
  mu <- rep(NA,m)

  #Gibbs sampler
  for (i in 1:numsamp){
     # sampling mu's
     for (j in 1:m){
      curvar <- 1/(n[j]/sigsq + 1/tausq)
          curmean <- (means[j]*n[j]/sigsq + mu0/tausq)*curvar
          mu[j] <- rnorm(1,mean=curmean,sd=sqrt(curvar))
     }
    
     # sampling mu0
     #mu0 <- rnorm(1,mean=mean(mu),sd=sqrt(tausq/m))
     mu0 <- rgamma(1,shape=sqrt(tausq/m), rate=sqrt(tausq/m)/mean(mu))

    # sampling tausq
     sumsq.mu <- sum((mu-mu0)^2)
     tausqinv <- rgamma(1,shape=((m-1)/2),rate=(sumsq.mu/2))
     tausq <- 1/tausqinv
     # sampling sigsq
     sumsq.y <- 0
     for (j in 1:m){
        district <- df.district$District[j]
        y <- df.fc%>% filter(District == district)%>%select(CullLag)%>%na.omit()
        sumsq.y <- sumsq.y + sum((y-mu[j])^2)
     }
      sigsqinv <- rgamma(1,shape=(ntot/2),rate=(sumsq.y/2))

      sigsq <- (1/sigsqinv)
      

     # storing sampled values
     mu.samp[i,] <- mu
     mu0.samp[i] <- mu0
     tausq.samp[i] <- tausq
     sigsq.samp[i] <- sigsq
  }
  
  #store to global env
  mu.samp <<- mu.samp
  parm.samp <<- data.frame(mu0.samp = mu0.samp,
                          tausq.samp = tausq.samp,
                          sigsq.samp = sigsq.samp)

}
```

## Model implementation

To implement a Gibbs sampler, starting values of \(\mu\_0\), \(\tau^2\), \(\sigma^2\) were arbitrarily selected and
then these parameters as well as \(\mu\_j\) were iteratively updated.

### Confirm convergence

Two chains were run with different arbitrary starting parameters
(chain 1: \(\mu\_0 = 1, \tau^2 =1, \sigma^2 =
1\), chain2:\(\mu\_0 = 20, \tau^2 =10,
\sigma^2 = 10\)) and plotted to check that chains converge
(Figure 3). For all parameters, the two chains did converge.

```
#RUN GIBBS SAMPLER 
#function: fun.gibbs(numsamp, sigsq, tausq, mu0, df)
fun.gibbs(1000, 1, 1, 1, df.fc); mu.samp1 <- mu.samp; parm.samp1 <- parm.samp #first chain
fun.gibbs(1000, 10, 10, 20, df.fc); mu.samp2 <- mu.samp; parm.samp2 <- parm.samp #second chain
```

**Figure 2: Global convergence
evaluation.**

After convergence was confirmed, burn in time was evaluated. The
first 100 samples of both chains were plotted to estimate a burn in time
(ie time until the chains converge). The first 20 samples were removed
as burn in.  
             
**Figure 3: Burn in evaluation.**

```
### throwing away first 20 as burn-in 
mu.samp1 <- mu.samp1[21:nrow(mu.samp1),]
mu.samp2 <- mu.samp2[21:nrow(mu.samp2),]
parm.samp1 <- parm.samp1[21:nrow(parm.samp1),]
parm.samp2 <- parm.samp2[21:nrow(parm.samp2),]
```

### Evaluate and eliminate autocorrelation

Both chains were evaluated for autocorrelation.

**Figure 4: Initial autocorrelation
evaluation.**

In order to produce independent samples, the chains were thinned to
remove autocorrelation: 1 out of every 10 samples was kept. After
thinning, autocorrelation was re-evaluatedto ensure non-autocorrelated
samples.

```
# thinning chains 
Thin <- 10
temp <- Thin*c(1:(nrow(mu.samp2)/Thin))
mu.samp1 <- mu.samp1[temp,]
mu.samp2 <- mu.samp2[temp,]
parm.samp1 <- parm.samp1[temp,]
parm.samp2 <- parm.samp2[temp,]
```

            
**Figure 5: Autocorrelation evaluation after
thinning.**

After thinning, chains were merged into a combined posterior sample
set.

```
#FINAL SAMPLE

#combine chains
mu.samp <- rbind(mu.samp1,mu.samp2)
parm.samp <- rbind(parm.samp1, parm.samp2)

# Calculate statistics
df.district = df.fc %>% select(id, District, CullLag)%>%
  group_by(District) %>%
  summarise(mean = mean(CullLag, na.rm=TRUE), n = n())

df.post <- data.frame(district = df.district$District, 
                      means=df.district$mean, 
                      n= df.district$n,
                      mu.postmean =apply(mu.samp,2,mean), 
                      mu0.postmean=mean(parm.samp$mu0.samp))%>%
  mutate(means = round(means, 2),
         mu.postmean = round(mu.postmean,2))
```

## Results

**Table 1: Means and 95% posterior intervals of
global parameters**

| Parameter | Mean | Lower | Upper |
| --- | --- | --- | --- |
| mu0 | 2.31 | 0.00 | 9.89 |
| sigma sq | 4.91 | 3.93 | 6.14 |
| tau sq | 14.79 | 1.48 | 78.20 |

**Figure 7: Histograms of global
parameters**

The modeled \(\mu\)’s were then
compared to the data means. Due to the hierarchical model nature, the
means with less data supporting it, such as the case of the Jose Luis
Bustamante y R. district were pulled dramatically towards the global
mean, while districts with a lot of data support, such as the district
of Cerro Colorado were barely budged.

**Figure 7: Shrinkage of normal
means.**

**Table 2: modeled means versus data
means**

| District | Data mean | number of observations | Posterior mean, \(\mu\_j\) |
| --- | --- | --- | --- |
| ALTO SELVA ALEGRE | 3.00 | 3 | 2.73 |
| AREQUIPA | 0.00 | 2 | 0.66 |
| CAYMA | 1.78 | 9 | 1.81 |
| CERRO COLORADO | 2.35 | 123 | 2.33 |
| CHARACATO | 6.00 | 2 | 4.77 |
| CIUDAD DE DIOS | 2.33 | 3 | 2.19 |
| HUNTER | 1.50 | 4 | 1.49 |
| JOSE LUIS BUSTAMANTE Y RIVERO | 8.00 | 1 | 5.13 |
| MARIANO MELGAR | 2.00 | 9 | 2.01 |
| MIRAFLORES | 0.67 | 3 | 0.79 |
| MOLLEBAYA | 2.50 | 4 | 2.33 |
| PAUCARPATA | 1.75 | 4 | 1.85 |
| PEDREGAL | 3.00 | 9 | 2.85 |
| QUEQUEÑA | 1.00 | 1 | 1.26 |
| SOCABAYA | 3.75 | 4 | 3.45 |
| YURA | 2.88 | 16 | 2.87 |

## Model Evaluation

The hierarchical model was evaluated using a Bayesian posterior
predictive check. To evaluate whether the observed data look extreme
compared to data generated from the model, a test statistic not directly
evaluated by the model was selected - max(\(\mu\_j\)). The maximum district mean, \(\mu\_j\), observed was compared to the
simulated maximum \(\mu\_j\)’s. The
observed maximum is not significantly extreme indicating a reasonable
model, especially considering an important property of the hierarchical
model is to shrink extreme values that are not supported by a large
sample.

```
## Test statistic: max of mu_j from observed data
test.data <- max(df.district$mean)

# max mu_j's from simulated samples
max.mu.samp <- NULL
for(i in 1:nrow(mu.samp)){
  max <- max(mu.samp[i,])
  max.mu.samp <- rbind(max.mu.samp, max)
}

test2.p <- sum(max.mu.samp > test.data)/nrow(mu.samp)
```

**Figure 8: The distribution of simulated maximum
\(mu\_j\) with the observed maximum
\(mu\_j\) indicated by the red
line.**
