# Supplementary material for: Challenging a paradigm: Staggered versus single-pulse mass dog vaccination strategy for rabies elimination
Source: PLoS Comput Biol. 2025 Feb 7;21(2):e1012780. doi: 10.1371/journal.pcbi.1012780 (PMC11805426; doi:10.1371/journal.pcbi.1012780)
Supplement: S3 Text — Base map and data from OpenStreetMap and OpenStreetMap Foundation (26,27). HTML file can be opened in any web browser. (HTML) [file pcbi.1012780.s003.html]

Microred visualization


# Microred visualization

#### B. Bellotti

#### 10/21/2024

### Load data

```
sf.microred <- st_read(here::here("data_minimal", "sf.microred.shp"), quiet=TRUE)
sf.dist<-st_read(here::here("data_minimal", "microred.shp"), quiet=TRUE)

load(here::here("data_minimal", "df.MR.parms.Rda"))

df.MR.parms <- df.MR.parms%>%
  rename(Microred = microred)

sf.MR <- left_join(sf.microred, df.MR.parms)
```

### Visualize microreds

```
pal <- colorBin("YlOrRd", domain = sf.MR$N, 
                bins = c(0, 7500, 15000, 30000, 60000))

leaflet() %>%
  setView(lng = -71.54, lat = -16.41, zoom = 11)%>% #center in AQP
  addProviderTiles(providers$CartoDB.Positron)%>% # add third party provider tile
  addPolygons(data=sf.MR, color = "black", fillOpacity = 0.75, fillColor = ~pal(N))%>%
  addScaleBar()%>%
  addLegend("bottomright", data= sf.MR,
              pal=pal,
              values=~N,    
              title = 'Dog population estimates',
              opacity = 0.8)
```

### Inverse distance from centroid

```
### visuaize ########
pal <- colorNumeric(
  palette = "Blues",
  domain = sf.dist$dist,
  reverse=TRUE)


leaflet() %>%
  setView(lng = -71.54, lat = -16.41, zoom = 11)%>% #center in AQP
  addProviderTiles(providers$CartoDB.Positron)%>% # add third party provider tile
  addPolygons(data=sf.microred, color= "black", fill=NA)%>%
  addPolylines(data=sf.dist, color=~pal(dist))%>%
  addScaleBar()
```

### Dog density frequency histogram

```
sf_use_s2(FALSE)
sf.MR$area = st_area(sf.MR)/1e+6 #units = km^2
sf.MR <- sf.MR %>%
  mutate(density = N/area)%>%
  units::drop_units()

ggplot()+
  theme_classic()+
  theme(text=element_text(size=24))+
  geom_histogram(data=sf.MR, aes(x= density), bins=8, color="black", fill="gray")+
  xlab("Dog density per sq. km") + ylab("Number of microreds")
```
