# Supplementary material for: Challenging a paradigm: Staggered versus single-pulse mass dog vaccination strategy for rabies elimination
Source: PLoS Comput Biol. 2025 Feb 7;21(2):e1012780. doi: 10.1371/journal.pcbi.1012780 (PMC11805426; doi:10.1371/journal.pcbi.1012780)
Supplement: S4 Text — HTML file can be opened in any web browser. (HTML) [file pcbi.1012780.s004.html]

Vaccination Scenario Simulations


# Vaccination Scenario Simulations

#### B. Raynor

#### 5/12/2022

### Set up

Load packages, functions, data

```
#clear environment
rm(list = ls()) 

#load required packages
library(ggplot2)  #pretty figures
library(dplyr)    #tidy code
library(ggpubr)   #plot arrangement
library(viridis)  #good color palettes

#load source functions
# source("Functions_InputFormatting.R")
source(here::here("R", "Functions_StochasticModels.R"))
source(here::here("R", "Functions_StochasticSimulationVisualization.R"))

#load parameterization data
#load("~/RabiesLabPeru/modelling_rabies/06_SPatialHeterogeneity/Parameters/df.contact.Rda")
#load("~/RabiesLabPeru/modelling_rabies/06_SPatialHeterogeneity/Parameters/df.parms.Rda")
#load("~/RabiesLabPeru/modelling_rabies/06_SPatialHeterogeneity/Parameters/df.MR.parms.Rda")
#load("~/RabiesLabPeru/modelling_rabies/06_SpatialHeterogeneity/Parameters/df.MR.contact.Rda")
load(here::here("data_minimal", "df.MR.parms.Rda")) 
load(here::here("data_minimal", "df.MR.contact.Rda")) 

df.parms <- df.MR.parms%>%
  mutate(current_infect = c(0,1,0,0,0,1,1,1,0,0,0,0,0,1,0,1,1,0,1,0), #current 2018
         alpha1 = c(1.85, 2.73,1.85,1.78, 1.85, 2.87,
                    0.79, 1.81, 2.01, 1.49, 2.01,3.45,
                    2.31, 5.13, 2.31, 2.33, 2.33, 
                    4.77, 2.33, 2.33),
         alpha= 1/alpha1)

df.parms <- df.parms %>%
  mutate(b=10)

df.contact <- df.MR.contact/max(df.MR.contact)
```

Test for tracker variable - delete this section if doesn’t work
Rewrite functions to have a tracker variable

```
rfun.PatchSEIV <- function(init, parms, beta.contact, vax, Time, dt){
  #list frame to hold num individuals in each state in each sim
  
  T= Time/dt 
  
  X <- init%>%
    rfun.FormatInit()
  
  Hinit <- t(rep(0, num_patch))
  X <- cbind(X, Hinit)
  
  times <- c(seq(dt, Time, by=dt))
  
  #loop through each step of approximation
  for(q in 1:T){ #check to see if you want to write this as second step or based of init values
    
    #current time point
    current_time <- times[q]
    X1 <- X[nrow(X),] # call up last time point
    X1[1, (4*num_patch+1):(5*num_patch)] =0 #reset tracker 
    
    #current N
    N = (rep(0,num_patch)) #create empty frame
    for(j in 1:num_patch){
      for(i in 0:(num_state-1)){
        N[j] = N[j] + X1[j + (i*num_patch)][,1] 
      }
    } 
    
    
    #Pull apart states
    S= as.matrix(t(X1[1, 1:num_patch]))
    E= as.matrix(t(X1[1, (num_patch+1):(2*num_patch)]))
    I= as.matrix(t(X1[1, (2*num_patch+1):(3*num_patch)]))
    V= as.matrix(t(X1[1, (3*num_patch+1):(4*num_patch)]))
    #H = as.matrix(t(X1[1, (4*num_patch+1):(5*num_patch)]))
    
    #Pull apart parms
    beta = parms$beta
    b = parms$b
    mu =parms$mu
    gamma = parms$gamma
    alpha = parms$alpha
    nu2 <- vax[current_time,] #subset to current vax time
    #nu2 <- inst.vac(nu2)
    
    
    #Terms used
    bSI = beta*S*I
    bSI.contact = beta.contact%*%I*S
    birth.rate = b*N*(1-N/K) #carrying capacity birth rate
    birth.rate = ifelse(birth.rate > 0, birth.rate, 0) #no negative births
    
    #Create tracker variable
    H.vector = X
    H.vector[1,] =0
    H.vector[1, (4*num_patch+1):(5*num_patch)] = gamma*E
    H.final <- t(H.vector[1, (4*num_patch+1):(5*num_patch)])
    
    H_final <-  t(H.vector[1, (4*num_patch+1):(5*num_patch)])
    H_final <- floor(H_final)
    
    #10% chance of report
    for (f in 1:length(H_final)){
      f1 <- H_final[f]
      H_final[f] = rbinom(1,f1,.8) #prob of detection
      }

    
    #System of equations
    dS <- birth.rate -bSI - bSI.contact - nu2*S - mu*S
    dE <- bSI + bSI.contact -gamma*E - mu*E
    dI <- gamma*E - alpha*I - mu*I
    dV <- nu2*S-mu*V

    #abs value of all terms in each state
    S_terms = birth.rate  + bSI + bSI.contact + nu2*S + mu*S
    E_terms = bSI + bSI.contact + gamma*E + mu*E
    I_terms = gamma*E + alpha*I + mu*I
    V_terms = nu2*S + mu*V
    
    #Expected values
    Ex.X <- rbind(dS, dE, dI, dV)
    
    #Covariance matrix
    V11 = matrix(diag(S_terms[,1]), ncol=num_patch)
    V21 = beta.contact*matrix(rep(I, num_patch), ncol=num_patch)*
      matrix(rep(S, num_patch), ncol=num_patch, byrow=TRUE) + 
      matrix(diag(bSI[,1]), ncol=num_patch)
    V31 = matrix(rep(0, num_patch^2), nrow=num_patch)
    V41 = matrix(diag(nu2*S[,1]), ncol=num_patch) #FIX THIS WITH APPROX FUN
    
    V12 = t(V21)
    V22 = matrix(diag(E_terms[,1]), ncol=num_patch)
    V32 = matrix(diag(gamma*E[,1]), ncol=num_patch)
    V42 = matrix(rep(0, num_patch^2), nrow=num_patch)
    
    V13 = t(V31)
    V23 = t(V32)
    V33 = matrix(diag(I_terms[,1]), ncol=num_patch)
    V43 = matrix(rep(0, num_patch^2), nrow=num_patch)
    
    V14 = t(V41)
    V24 = t(V42)
    V34 = t(V43)
    V44 = matrix(diag(V_terms[,1]), ncol=num_patch)
    
    
    V = cbind(rbind(V11, V21, V31, V41),
              rbind(V12, V22, V32, V42),
              rbind(V13, V23, V33, V43),
              rbind(V14, V24, V34, V44))
    
    C=sqrt(V) 
    
    #############################################################################
    #Calculate population at next step
    #############################################################################
    #Create a random vector for each population
    ra = matrix(rnorm(num_comps^2, mean=0, sd=1), nrow = num_comps)
    
    #Sum cov for next step
    F_sum = rowSums((ra*C))
    state_change = dt*Ex.X + F_sum*sqrt(dt)
    # state_change =c(state_change, t(H.vector[1, (4*num_patch+1):(5*num_patch)]))
    state_change =c(state_change, H_final)
    
    #Caluclate the populations at the next step
    X2 <- X1 + state_change
    X2 <- ifelse(X2 > 0.5, X2, 0)
    X <- rbind(X, X2)
    
    
  } #end of big loop
  out <- data.frame(time=seq(0, Time, by=dt), X)
  return(out)
}
```

Initialize SDEs

```
#parameters
parms <- df.parms %>%
  mutate(beta=0.00001)


#contact matrix
beta.contact_constant = 0.00001 
beta.contact = rfun.FormatContact(df.contact, beta.contact_constant)

num_patch = length(df.contact)

#initial conditions 
init <- data.frame(S=0.5*df.parms$N - 100*df.parms$current_infect,
                   E= rep(0,num_patch),
                   I= 100*df.parms$current_infect,
                   V= 0.5*df.parms$N)

#time series
num_years = 6
Time = 365*num_years #End time
dt = 1 #Step size dt

#How many simulations we want to average
sim=100

#pulse vax
vax0= matrix(0, Time/dt, num_patch)
```

## Simulation 1: single pulse vaccination all 50% yearly x3 years

```
set.seed(123)

#set up VANCAN pulse schedules
vax <- vax0
vax[c(100, (100+365), (100+2*365), (100+3*365), (100+4*365), (100+5*365)),] = c(rep(0.5, num_patch)) #pulse of 0.8 coverage at time points 1, 10

#save simulations together in a big df
out <- NULL
for(i in 1:sim){
  sub <-  rfun.PatchSEIV(init, parms, beta.contact, vax, Time, dt)%>%
    mutate(sim = i)
  out <- rbind(out, sub)
}

rfun_RabiesDynamics(out, df.parms$microred)
```

All states

Just transmission states

All infected districts + total on one plot

avg time of elimination

```
dfS <- out

#find total outbreak size
dfS$X <- dfS%>%
  select(X1:X20)%>%
  rowSums()

dfS0 <- dfS%>%
  select(time, sim, X)%>%
  group_by(sim)%>%
  summarize(total = sum(X))

knitr::kable(dfS0%>% summarise(SI_2.5 = quantile(total, .025), 
                               SI_97.5 = quantile(total, .975),
                               mean = round(mean(total))),
                         caption = "Simulated cases")
```

Simulated cases

| SI\_2.5 | SI\_97.5 | mean |
| --- | --- | --- |
| 2675.025 | 8470.425 | 4897 |

```
#find time to elimination
dfS$I <- dfS%>%
  select(I1:I20)%>%
  rowSums()

dfS <- dfS %>%
  select(time, sim, I)%>%
  filter(I != 0)%>%
  group_by(sim)%>%
  summarize(max = max(time))

dfS <- dfS%>%
  mutate(Elim = ifelse(max==2190, "no", "yes"))%>%
  filter(Elim == "yes")
  
dfS1 <- dfS %>%
  summarise(SI_2.5 = quantile(max, .025),
            SI_97.5 = quantile(max, .975),
            mean = round(mean(max)))%>%
  mutate(Prop_elim = length(dfS$Elim)/sim)

knitr::kable(dfS1,
            caption = "Avg time to elimination if eliminated, Proportion eliminated")
```

Avg time to elimination if eliminated, Proportion
eliminated

| SI\_2.5 | SI\_97.5 | mean | Prop\_elim |
| --- | --- | --- | --- |
| 1041 | 2126.2 | 1520 | 0.97 |

## Simulation 2: single pulse vaccination 80%, yearly for 6 years

```
set.seed(123)

#set up VANCAN pulse schedules
vax <- vax0
vax[c(100, (100+365), (100+2*365), (100+3*365), (100+4*365), (100+5*365)),] = c(rep(0.8, num_patch)) #pulse of 0.8 coverage at time points 1, 10

#save simulations together in a big df
out <- NULL
for(i in 1:sim){
  sub <-  rfun.PatchSEIV(init, parms, beta.contact, vax, Time, dt)%>%
    mutate(sim = i)
  out <- rbind(out, sub)
}

rfun_RabiesDynamics(out, df.parms$microred)
```

All states

Just transmission states

All infected districts + total on one plot

avg time of elimination

```
dfS <- out

#find total outbreak size
dfS$X <- dfS%>%
  select(X1:X20)%>%
  rowSums()

dfS0 <- dfS%>%
  select(time, sim, X)%>%
  group_by(sim)%>%
  summarize(total = sum(X))

knitr::kable(dfS0%>% summarise(SI_2.5 = quantile(total, .025), 
                               SI_97.5 = quantile(total, .975),
                               mean = round(mean(total))),
                         caption = "Simulated cases")
```

Simulated cases

| SI\_2.5 | SI\_97.5 | mean |
| --- | --- | --- |
| 1052.275 | 2519.625 | 1812 |

```
#find time to elimination
dfS$I <- dfS%>%
  select(I1:I20)%>%
  rowSums()

dfS <- dfS %>%
  select(time, sim, I)%>%
  filter(I != 0)%>%
  group_by(sim)%>%
  summarize(max = max(time))

dfS <- dfS%>%
  mutate(Elim = ifelse(max==2190, "no", "yes"))%>%
  filter(Elim == "yes")
  
dfS1 <- dfS %>%
  summarise(SI_2.5 = quantile(max, .025),
            SI_97.5 = quantile(max, .975),
            mean = round(mean(max)))%>%
  mutate(Prop_elim = length(dfS$Elim)/sim)

knitr::kable(dfS1,
            caption = "Avg time to elimination if eliminated, Proportion eliminated")
```

Avg time to elimination if eliminated, Proportion
eliminated

| SI\_2.5 | SI\_97.5 | mean | Prop\_elim |
| --- | --- | --- | --- |
| 425.85 | 716.175 | 567 | 1 |

# STAGGERED

## Simulation 3: staggered vaccination all 50%

```
set.seed(123)

#set up VANCAN pulse schedules
vax <- vax0
vax_coverage = 0.5

vax[c(253, (253+365), (253+2*365), (253+3*365), (253+4*365), (253+5*365)), 1] = vax_coverage
vax[c(211, (211+365), (211+2*365), (211+3*365), (211+4*365), (211+5*365)), 2] = vax_coverage
vax[c(267, (267+365), (267+2*365), (267+3*365), (267+4*365), (267+5*365)), 3] = vax_coverage
vax[c(134, (134+365), (134+2*365), (134+3*365), (134+4*365), (134+5*365)), 4] = vax_coverage
vax[c(260, (260+365), (260+2*365), (260+3*365), (260+4*365), (260+5*365)), 5] = vax_coverage
vax[c(155, (155+365), (155+2*365), (155+3*365), (155+4*365), (155+5*365)), 6] = vax_coverage
vax[c(232, (232+365), (232+2*365), (232+3*365), (232+4*365), (232+5*365)), 7] = vax_coverage
vax[c(141, (141+365), (141+2*365), (141+3*365), (141+4*365), (141+5*365)), 8] = vax_coverage
vax[c(240, (240+365), (240+2*365), (240+3*365), (240+4*365), (240+5*365)), 9] = vax_coverage
vax[c(169, (169+365), (169+2*365), (169+3*365), (169+4*365), (169+5*365)), 10] = vax_coverage
vax[c(240, (240+365), (240+2*365), (240+3*365), (240+4*365), (240+5*365)), 11] = vax_coverage
vax[c(162, (162+365), (162+2*365), (162+3*365), (162+4*365), (162+5*365)), 12] = vax_coverage
vax[c(190, (190+365), (190+2*365), (190+3*365), (190+4*365), (190+5*365)), 13] = vax_coverage
vax[c(173, (173+365), (173+2*365), (173+3*365), (173+4*365), (173+5*365)), 14] = vax_coverage
vax[c(197, (197+365), (197+2*365), (197+3*365), (197+4*365), (197+5*365)), 15] = vax_coverage
vax[c(281, (281+365), (281+2*365), (281+3*365), (281+4*365), (281+5*365)), 16] = vax_coverage
vax[c(281, (281+365), (281+2*365), (281+3*365), (281+4*365), (281+5*365)), 17] = vax_coverage
vax[c(274, (274+365), (274+2*365), (274+3*365), (274+4*365), (274+5*365)), 18] = vax_coverage
vax[c(288, (288+365), (288+2*365), (288+3*365), (288+4*365), (288+5*365)), 19] = vax_coverage
vax[c(322, (322+365), (322+2*365), (322+3*365), (322+4*365), (322+5*365)), 20] = vax_coverage


#save simulations together in a big df
out <- NULL
for(i in 1:sim){
  sub <-  rfun.PatchSEIV(init, parms, beta.contact, vax, Time, dt)%>%
    mutate(sim = i)
  out <- rbind(out, sub)
}

rfun_RabiesDynamics(out, df.parms$microred)
```

All states

Just transmission states

All infected districts + total on one plot

stats

```
dfS <- out

#find total outbreak size
dfS$X <- dfS%>%
  select(X1:X20)%>%
  rowSums()

dfS0 <- dfS%>%
  select(time, sim, X)%>%
  group_by(sim)%>%
  summarize(total = sum(X))

knitr::kable(dfS0%>% summarise(SI_2.5 = quantile(total, .025), 
                               SI_97.5 = quantile(total, .975),
                               mean = round(mean(total))),
                         caption = "Simulated cases")
```

Simulated cases

| SI\_2.5 | SI\_97.5 | mean |
| --- | --- | --- |
| 15582.15 | 40073.53 | 26850 |

```
#find time to elimination
dfS$I <- dfS%>%
  select(I1:I20)%>%
  rowSums()

dfS <- dfS %>%
  select(time, sim, I)%>%
  filter(I != 0)%>%
  group_by(sim)%>%
  summarize(max = max(time))

dfS <- dfS%>%
  mutate(Elim = ifelse(max==2190, "no", "yes"))%>%
  filter(Elim == "yes")
  
dfS1 <- dfS %>%
  summarise(SI_2.5 = quantile(max, .025),
            SI_97.5 = quantile(max, .975),
            mean = round(mean(max)))%>%
  mutate(Prop_elim = length(dfS$Elim)/sim)

knitr::kable(dfS1,
            caption = "Avg time to elimination if eliminated, Proportion eliminated")
```

Avg time to elimination if eliminated, Proportion
eliminated

| SI\_2.5 | SI\_97.5 | mean | Prop\_elim |
| --- | --- | --- | --- |
| 1545.825 | 2188.225 | 1952 | 0.72 |

## Simulation 4: staggered vaccination all 80%

```
#set up VANCAN pulse schedules
vax <- vax0
vax_coverage = 0.8

vax[c(253, (253+365), (253+2*365), (253+3*365), (253+4*365), (253+5*365)), 1] = vax_coverage
vax[c(211, (211+365), (211+2*365), (211+3*365), (211+4*365), (211+5*365)), 2] = vax_coverage
vax[c(267, (267+365), (267+2*365), (267+3*365), (267+4*365), (267+5*365)), 3] = vax_coverage
vax[c(134, (134+365), (134+2*365), (134+3*365), (134+4*365), (134+5*365)), 4] = vax_coverage
vax[c(260, (260+365), (260+2*365), (260+3*365), (260+4*365), (260+5*365)), 5] = vax_coverage
vax[c(155, (155+365), (155+2*365), (155+3*365), (155+4*365), (155+5*365)), 6] = vax_coverage
vax[c(232, (232+365), (232+2*365), (232+3*365), (232+4*365), (232+5*365)), 7] = vax_coverage
vax[c(141, (141+365), (141+2*365), (141+3*365), (141+4*365), (141+5*365)), 8] = vax_coverage
vax[c(240, (240+365), (240+2*365), (240+3*365), (240+4*365), (240+5*365)), 9] = vax_coverage
vax[c(169, (169+365), (169+2*365), (169+3*365), (169+4*365), (169+5*365)), 10] = vax_coverage
vax[c(240, (240+365), (240+2*365), (240+3*365), (240+4*365), (240+5*365)), 11] = vax_coverage
vax[c(162, (162+365), (162+2*365), (162+3*365), (162+4*365), (162+5*365)), 12] = vax_coverage
vax[c(190, (190+365), (190+2*365), (190+3*365), (190+4*365), (190+5*365)), 13] = vax_coverage
vax[c(173, (173+365), (173+2*365), (173+3*365), (173+4*365), (173+5*365)), 14] = vax_coverage
vax[c(197, (197+365), (197+2*365), (197+3*365), (197+4*365), (197+5*365)), 15] = vax_coverage
vax[c(281, (281+365), (281+2*365), (281+3*365), (281+4*365), (281+5*365)), 16] = vax_coverage
vax[c(281, (281+365), (281+2*365), (281+3*365), (281+4*365), (281+5*365)), 17] = vax_coverage
vax[c(274, (274+365), (274+2*365), (274+3*365), (274+4*365), (274+5*365)), 18] = vax_coverage
vax[c(288, (288+365), (288+2*365), (288+3*365), (288+4*365), (288+5*365)), 19] = vax_coverage
vax[c(322, (322+365), (322+2*365), (322+3*365), (322+4*365), (322+5*365)), 20] = vax_coverage
#save simulations together in a big df
out <- NULL
for(i in 1:sim){
  sub <-  rfun.PatchSEIV(init, parms, beta.contact, vax, Time, dt)%>%
    mutate(sim = i)
  out <- rbind(out, sub)
}

rfun_RabiesDynamics(out, df.parms$microred)
```

All states

Just transmission states

All infected districts + total on one plot

avg time of elimination

```
dfS <- out

#find total outbreak size
dfS$X <- dfS%>%
  select(X1:X20)%>%
  rowSums()

dfS0 <- dfS%>%
  select(time, sim, X)%>%
  group_by(sim)%>%
  summarize(total = sum(X))

knitr::kable(dfS0%>% summarise(SI_2.5 = quantile(total, .025), 
                               SI_97.5 = quantile(total, .975),
                               mean = round(mean(total))),
                         caption = "Simulated cases")
```

Simulated cases

| SI\_2.5 | SI\_97.5 | mean |
| --- | --- | --- |
| 6051 | 13167.6 | 9324 |

```
#find time to elimination
dfS$I <- dfS%>%
  select(I1:I20)%>%
  rowSums()

dfS <- dfS %>%
  select(time, sim, I)%>%
  filter(I != 0)%>%
  group_by(sim)%>%
  summarize(max = max(time))

dfS <- dfS%>%
  mutate(Elim = ifelse(max==2190, "no", "yes"))%>%
  filter(Elim == "yes")
  
dfS1 <- dfS %>%
  summarise(SI_2.5 = quantile(max, .025),
            SI_97.5 = quantile(max, .975),
            mean = round(mean(max)))%>%
  mutate(Prop_elim = length(dfS$Elim)/sim)

knitr::kable(dfS1,
            caption = "Avg time to elimination if eliminated, Proportion eliminated")
```

Avg time to elimination if eliminated, Proportion
eliminated

| SI\_2.5 | SI\_97.5 | mean | Prop\_elim |
| --- | --- | --- | --- |
| 710.85 | 966.3 | 826 | 1 |
